# Global job satisfaction and fluctuation among community general practitioners: a systematic review and meta-analysis

**DOI:** 10.1186/s12913-024-10792-9

**Published:** 2024-03-27

**Authors:** Qilin Deng, Yifang Liu, Ziyi Cheng, Qi Wang, Junan Liu

**Affiliations:** 1 Department of Social Medicine and Health Management, School of Public Health, Tongji Medical College, Huazhong University of Science and Technology, 430030 Wuhan, China; 2 Department of Epidemiology and Biostatistics, School of Public Health, Tongji Medical College, Huazhong University of Science and Technology, 430030 Wuhan, China

**Keywords:** Job satisfaction, General practitioners, Influencing factor, Meta-analysis

## Abstract

**Introduction:**

Community General Practitioners (CGPs) are crucial to primary healthcare worldwide. Their job satisfaction significantly impacts the quality and accessibility of healthcare. However, a comprehensive global perspective on this issue remains absent, necessitating this systematic review and meta-analysis.

**Methods:**

This systematic review and meta-analysis sourced literature from PubMed, Web of Science, CNKI, and Wanfang, up to June 14, 2023. Of the 2,742 identified studies, 100 articles were selected for meta-analysis to assess satisfaction levels, and 97 studies were chosen for comparative analysis of influential factors. We employed both meta-analytic and comparative analytic methodologies, focusing on varying geographical, economic, and temporal contexts.

**Results:**

The pooled rate and corresponding 95% confidence interval (*CI*) for job satisfaction among CGPs was 70.82% (95%*CI*: 66.62–75.02%) globally. Studies utilizing 5-point score scale obtained a random effect size of 3.52 (95%*CI*: 3.43–3.61). Diverse factors influenced satisfaction, with remuneration and working conditions being predominant. A noticeable decline in job satisfaction has been observed since the coronavirus disease 2019 outbreak, with satisfaction rates dropping from an average of 72.39% before 2009 to 63.09% in those published after 2020.

**Conclusions:**

The downward trend in CGPs’ job satisfaction is concerning and warrants urgent attention from policymakers, especially in regions with an acute shortage of CGPs. The findings from this comprehensive review and meta-analysis provide essential insights for informed healthcare policy-making. It highlights the urgency of implementing strategies to enhance CGP satisfaction, thereby improving the effectiveness of primary healthcare systems globally.

**Supplementary Information:**

The online version contains supplementary material available at 10.1186/s12913-024-10792-9.

## Key message

**What is already known?**

Community general practitioners (CGPs) are integral to primary healthcare across the world. Existing studies on CGPs’ job satisfaction are region-specific, leaving a gap in a consolidated global perspective.

**What are the new findings?**

•This study presents the first meta-analysis on global CGPs’ job satisfaction, indicating a moderate general satisfaction level.

•Notable differences in satisfaction levels are observed across countries based on their economic conditions.

•There is a downward trend in global CGPs’ job satisfaction, indicating potential ongoing and systemic challenges.

**What do the new findings imply?**

The insights from this study provide a foundation for healthcare policymakers and community healthcare managers for informed strategic planning. The observed trends accentuate the necessity for targeted interventions and policy changes. Given the evidence, areas facing significant shortages in CGPs, like China, need to prioritize and recalibrate their strategies focused on CGP recruitment, retention, and well-being.

## Introduction

General practitioners (GPs), commonly known as family doctors, constitute the cornerstone of general medical services. The interpretation of this concept varies across different national contexts. In China, Community General Practitioners (CGPs) mainly refer to doctors who work in community health service stations or township health institutions to provide primary health care for residents. Elsewhere, the definition of GPs is close to the concepts of primary care physicians, family physicians in the United States and GPs in the United Kingdom (UK) [1, 2].

However, there is a global shortage of primary care professionals and a strong tendency to leave current posts [3]. In China, the shortage of CGPs is nearly 100,000 in community health service stations in urban areas [4], and the situation is even more serious in rural areas. In the European Union, the demand gap in healthcare workforce supply is projected to reach about 1 million by 2020, including a shortfall of 230,000 doctors [5]. Even in the UK, where CGPs are more established, recruitment is difficult and many vacancies exist [6]. Moreover, a significant proportion of CGPs are contemplating options such as early retirement or looking for some jobs with reduced clinical burden [7].

Factors that seriously affect the satisfaction level of CGPs include high levels of stress, low salaries, and heavy workloads [8]. Given the pivotal role of primary care in the healthcare system and its impact on public health, it becomes imperative to thoroughly investigate the current state of job satisfaction among CGPs and to explore the determinants influencing job satisfaction. Despite extensive research on CGPs’ job satisfaction, systematic studies on the job satisfaction status of CGPs worldwide are still lacking. On one hand, different surveys use various assessment scales to evaluate job satisfaction: Chinese studies mostly use the Minnesota studies Questionnaire (MSQ) short-form scale to evaluate job satisfaction, while other countries mostly use the Warr-Cook-Wall (WCW) job satisfaction scale to measure [9]. Although a large number of studies have been published, there is a lack of global systematic quantitative evaluation of CGPs’ satisfaction. On the other hand, the satisfaction level of CGPs varies with the development of health services, but with the absence of a global study of changes in GP satisfaction and the lack of in-depth analyses of the factors contributing to changes in satisfaction.

This systematic review and meta-analysis report the current status of job satisfaction of CGPs around the world and compare the differences in job satisfaction of CGPs in diverse regions, different levels of economic development, and time periods. It further clarifies the changes in the factors influencing the job satisfaction of global CGPs, provide policy ideas to improve the job satisfaction of CGPs in China, and provides basis for the development of policies on the attractiveness of CGPs.

## Methods

**Fig. 1:**
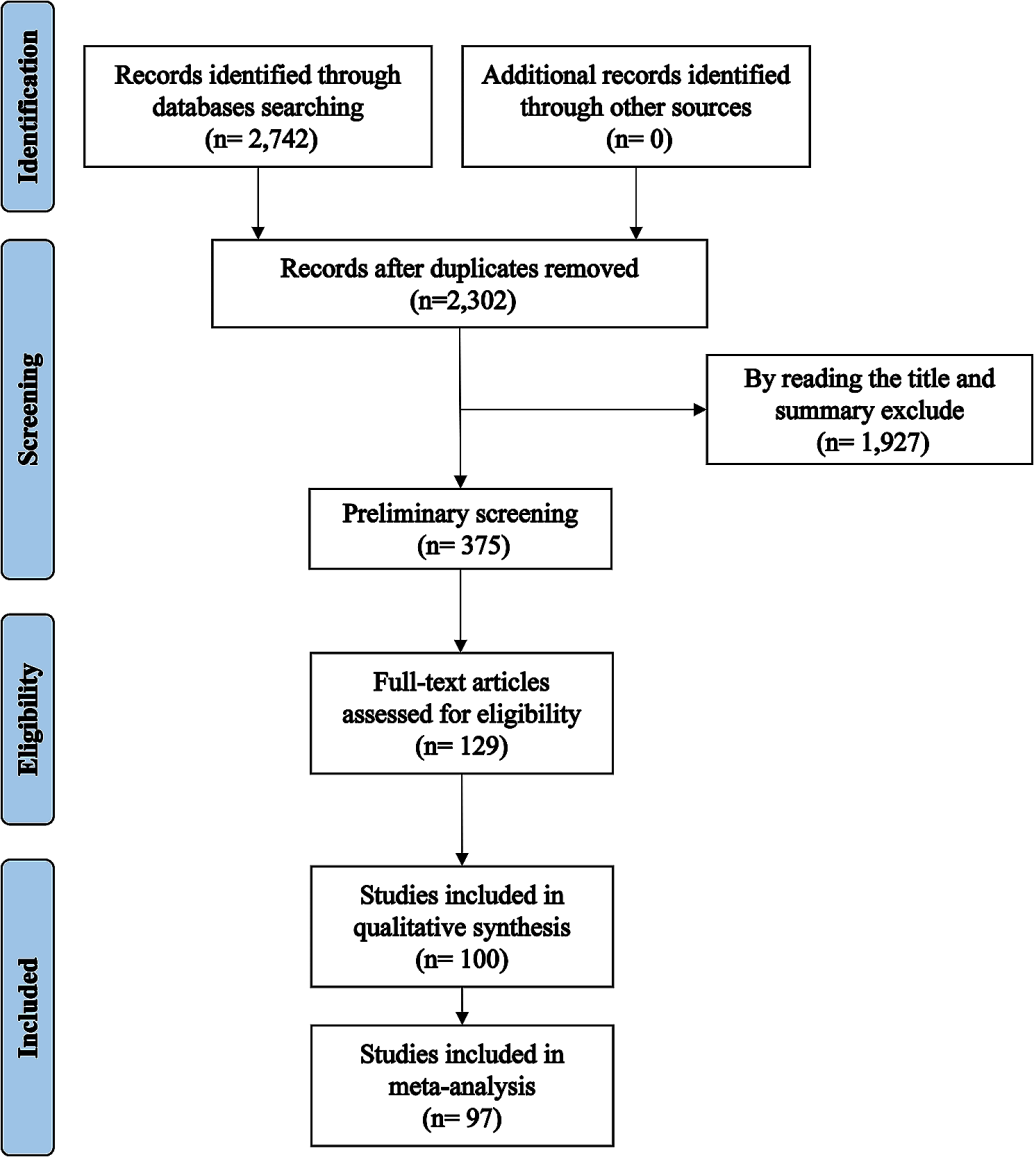
PRISMA flowchart of included studies

The protocol of this research was registered with PROSPERO (CRD42023421299) on 5 June 2023, and followed the Preferred Reporting Items for Systematic Reviews and Meta-Analyses (PRISMA) and Enhancing transparency in reporting the synthesis of qualitative research (ENTREQ) guidelines. (Fig. 1)

### Search strategy

We searched PubMed, China National Knowledge Infrastructure (CNKI), Web of Science, and Wan Fang databases, all with a search timeframe from database construction to 14 June 2023. The search strategy was based on the combination of subject terms and keywords, with “general practitioners”, “family physicians”, “job satisfaction” and other related phrases synthesized into the search strategy. (Supplementary 1).

### Study eligibility

Two researchers (Q.D. and Y.L.) chose potentially relevant articles from reviewed published literature. The eligibility criteria included the following: (1) studies with general practitioners; (2) studies that reported on job satisfaction or included factors influencing job satisfaction; and (3) observational study designs (cross-sectional, case-control, and cohort studies). The study excluded (1) specialists, nurses, and general medical students; (2) literature of a review nature; (3) duplicated literature by the same authors and with similar content; (4) literature with incomplete data; (5) inaccessible full-texts; and (6) literature not published in English or Chinese.

### Data extraction

Duplicates were first deleted using Zotero document management software and then screened according to the inclusion and exclusion criteria, divided into three steps. (1) Preliminary screening: Two reviewers excluded literature that did not meet the criteria based on the retrieved citation information, title and abstract. (2) Full-text review and rescreening: reading the literature, resolving disagreements through discussion, and excluding unqualified, such as incomplete data or inconsistency between the study object and the inclusion criteria literature. (3) Data extraction: this stage was performed on high-quality literature that met the criteria and reported the necessary data. (Supplementary 2).

### Quality assessment

The risk of potential bias was independently conducted by two evaluators (Q.D. and Y.L.). The assessment was grounded in the established criteria for appraising the quality of observational studies as outlined by the Centre for Evidence-Based Management’s Critical Appraisal Skill Program (CASP). Since one question was not relevant to our research, 11 items were used and combined with the evaluation methodology used in the study by Alicja Domagała et al. [7] to assess the methodological quality of the analysed literature, which was decided by a third reviewer (J.L.) in consultation when the two reviewers had different opinions. The total score was 11 and included studies were grouped into three categories according to the score: high quality (8–11), medium quality (4–7) and low quality (0–3).

### Data synthesis

After reviewing all the included literature, the collected studies were subjected to data information extraction into Excel software and narrative data synthesis: publication year, first author, the year studies were conducted, countries and regions where these studies performed, sampling method of the subjects, property of the institution (public or non-public), and influencing factors.

According to the World Bank’s 2022 classification of economies based on Gross National Income **(**GNI) per capita, national economies are classified as low-income, lower-middle-income, upper-middle-income, or high-income economies [10].

The results were presented as two sets of data: dichotomous data (i.e. the number of CGPs in the analysis who were satisfied or very satisfied in the whole group) and continuous data. To address the problem of high questionnaire heterogeneity in studies using diverse scales, job satisfaction as a continuous variable was converted to a common five-point scale: (1) “Very Dissatisfied”, (2) “Dissatisfied”, (3) “Neutral”, (4) “Satisfied”, (5) “Very Satisfied”. Satisfaction = reported score/maximum total score × 5.00. This formula converts satisfaction dimensions with varying total scores to a 5-point scale in the same proportion [11]. For example, for a questionnaire with a total score of 100, if the reported satisfaction score is 50, the converted score would be 50/100 × 5.00 = 2.50.

Meta-analysis was performed using R v 4.2.3 software and *P* < 0.05 was considered statistically significant. Data were tested for heterogeneity using *I*^2^ quantification and *Q*-tests. In detecting heterogeneity, if the heterogeneity of results across studies was caused by sampling error, the studies were considered homogeneous and a fixed-effects model was used; if the differences between the results of studies were beyond what could be explained by the sampling error (*I*^2^ > 50%), the random effect model was applied. Apparent heterogeneity was dealt with using subgroup and sensitivity analyses. Random effects models were conducted to test whether differences between groups were statistically significant and to explore possible sources of heterogeneity. Subgroup analyses were performed on data that could be extracted individually, such as the year the study was published, institutional attributes, sample size, region, and economic income level. Sensitivity analyses were conducted using a study-by-study approach to assess the robustness of the studies. Egger’s test combined with funnel plot was used to assess publication bias.

## Results

### Study characteristic

A total of 2,742 pieces of related literature were obtained from the initial screening. Duplicate publications and data duplication literature were excluded, and after the initial screening to read the title and abstract, and rescreening for full-text reading and quality assessment, 129 publications were finally included into the analysis, and the detailed screening process is shown in Fig. 1. After excluding publications with incomplete data and other non-conforming studies, 129 unique studies were finalized for inclusion, of which 68 articles provided data both included job satisfaction and related factors affecting job satisfaction, 29 provided only job satisfaction, and 32 only related factors on job satisfaction. Therefore, 97 articles were selected for meta-analysis of job satisfaction and 100 for conducting a comparative analysis of related factors. As the literature may report satisfaction results over multiple periods and with different sample sizes, there exists a single piece of literature that provides results from multiple studies for inclusion in the analysis, and of these 97 pieces of literature, 43 studies provided results in the form of dichotomous data, and 59 studies provided continuous data.

The main characteristics of the included studies are detailed in Table 1, while the basic information of the studies is detailed in the Supplementary 3. In this study, 60 studies (47.5%) were from middle-income countries, and 69 studies (53.5%) were from high-income countries. The number of studies from the Asian region accounted for 45.7% of the total, with 98 studies (76.0%) published after 2009. The quality assessment of the studies identified 68 studies (52.7%) as high quality and 61 studies (47.3%) as moderate quality. No low-quality articles was identified. A more detailed summary of the quality of the literature can be seen in the Supplementary 4.

**Table 1:** Characteristics of included studies

	Studies (*n* = 129)
Year of publication	
Before 2009	31 (24.0%)
2010–2019	60 (46.5%)
After 2020	38 (29.5%)
Country income level	
High income	69 (53.5%)
Upper-middle income	56 (43.4%)
Lower-middle income	4 (3.1%)
Region of study	
Asia	59 (45.7%)
Europe	43 (33.3%)
Middle East and Africa	6 (4.7%)
North America and Oceania	21 (16.3%)
Tools used to measure satisfaction	
WCW	41 (31.8%)
MSQ	31 (24.0%)
Others	57 (44.2%)
Quality assessment	
High	68 (52.7%)
Moderate	61 (47.3%)
Institutional Properties	
Public	74 (57.4%)
Non-public	20 (15.5%)
Not reported	35 (27.1%)

Note: WCW, Warr-Cook-Wall; MSQ, Minnesota studies Questionnaire;

### Overall job satisfaction

Forty-three studies provided satisfaction results in dichotomous data, i.e. the proportion of CGPs who were satisfied or very satisfied. The percentage of satisfied CGPs ranged from 26% up to 94% across studies. The random effect size of the CGPs’ job satisfaction score was 70.82% (95%CI: 66.62–75.02%), *I*^2^ = 99.3% and *Q* = 5798.51, *P* < 0.01 (Supplement 5). Based on a subjective examination of funnel diagram and Egger’s text (*P* > 0.05), there was no evidence of publication bias.

Fifty-nine studies provided satisfaction results in the form of continuous data, i.e., the average results of two scales used in the study, including 27 studies reported on a 5-point Likert scale and 31 studies reported on a 7-scale Likert scale. Converting the different dimensions of job satisfaction to a generic 5-point scale obtained a random effect size of 3.52 (95%CI: 3.43–3.61), *I*^2^ = 100%, *P* < 0.01. However, the Egger’s test showed a significant publication bias in overall job satisfaction (*P* = 0.01). Obvious asymmetry was also observed in the funnel plot (Supplement 6, 7). Consequently, the corrected results were achieved by trim-and-fill method and showed that the mean value of job satisfaction was 3.72 (95%CI: 3.62–3.82), *I*^2^ = 100%, *P* < 0.01.

### Subgroup analysis

For further examining the sources of study heterogeneity, subgroup analyses were conducted in the dichotomous data. As shown in Table 2, no significant differences in job satisfaction levels were found between study locations, organizational attributes, satisfaction measurement tools, urban and rural areas, and sample sizes. Instead, differences between groups were statistically significant (*P* < 0.05) in terms of income level and the time to publication of the articles in the study economies. Satisfaction among CGPs was lower in upper-middle-income countries 64.38% (95%CI: 57.11–71.65%) compared to high-income countries 74.49% (95%CI: 69.78–79.20%). The highest percentage of satisfied CGPs was 72.39% (95%CI: 65.42–79.35%) in studies conducted before 2009, while the lowest percentage of satisfied CGPs was 63.09% (95%CI: 60.68–65.49%) in studies conducted after 2020.

**Table 2:** Subgroup analysis of CGPs job satisfaction

	Reports (n)	Satisfied (%, 95%CI)	I^2^	*P* value for heterogeneity	*P* value between groups
Region of study					0.077
China	16	64.38 (57.11, 71.65)	98.50%	< 0.001	
Europe	12	71.39 (64.10, 78.69)	99.30%	< 0.001	
Middle East and Africa	3	72.31 (60.49, 84.12)	93.60%	< 0.001	
North America and Oceania	13	77.87 (70.82, 84.92)	99.40%	< 0.001	
Country income level					0.022
Upper-middle income	16	64.38 (57.11, 71.65)	98.50%	< 0.001	
High income	28	74.49 (69.78, 79.20)	99.30%	< 0.001	
Year of publication					0.004
Before 2009	12	72.39 (65.42, 79.35)	99.10%	< 0.001	
2010–2019	29	71.07 (65.43, 76.70)	99.30%	< 0.001	
After 2020	3	63.09 (60.68, 65.49)	0.00%	< 0.001	
Institutional Properties					0.171
Public	25	68.37 (62.32, 74.37)	99.10%	< 0.001	
Non-public	19	74.06 (68.53, 79.59)	99.30%	< 0.001	
Tools used to measure satisfaction					0.622
WCW	7	74.93 (62.78, 87.08)	99.50%	< 0.001	
MSQ	6	67.18 (57.36, 77.01)	96.30%	< 0.001	
Others	31	70.60 (65.58, 75.62)	99.20%	< 0.001	
Practice location					0.617
Urban	15	67.65 (59.33, 75.98)	98.70%	< 0.001	
Rural	6	71.93 (62.50, 81.36)	98.60%	< 0.001	
Mixed	23	72.60 (67.09, 78.10)	99.40%	< 0.001	
Sample size					0.442
< 500	19	68.22 (60.77, 75.66)	97.90%	< 0.001	
500–1000	6	75.83 (66.78, 84.88)	98.40%	< 0.001	
> 1000	19	71.72 (66.03, 77.41)	99.60%	< 0.001	

Note: CGPs, Community General Practitioners; WCW, Warr-Cook-Wall; MSQ, Minnesota studies Questionnaire

**Fig. 2:**
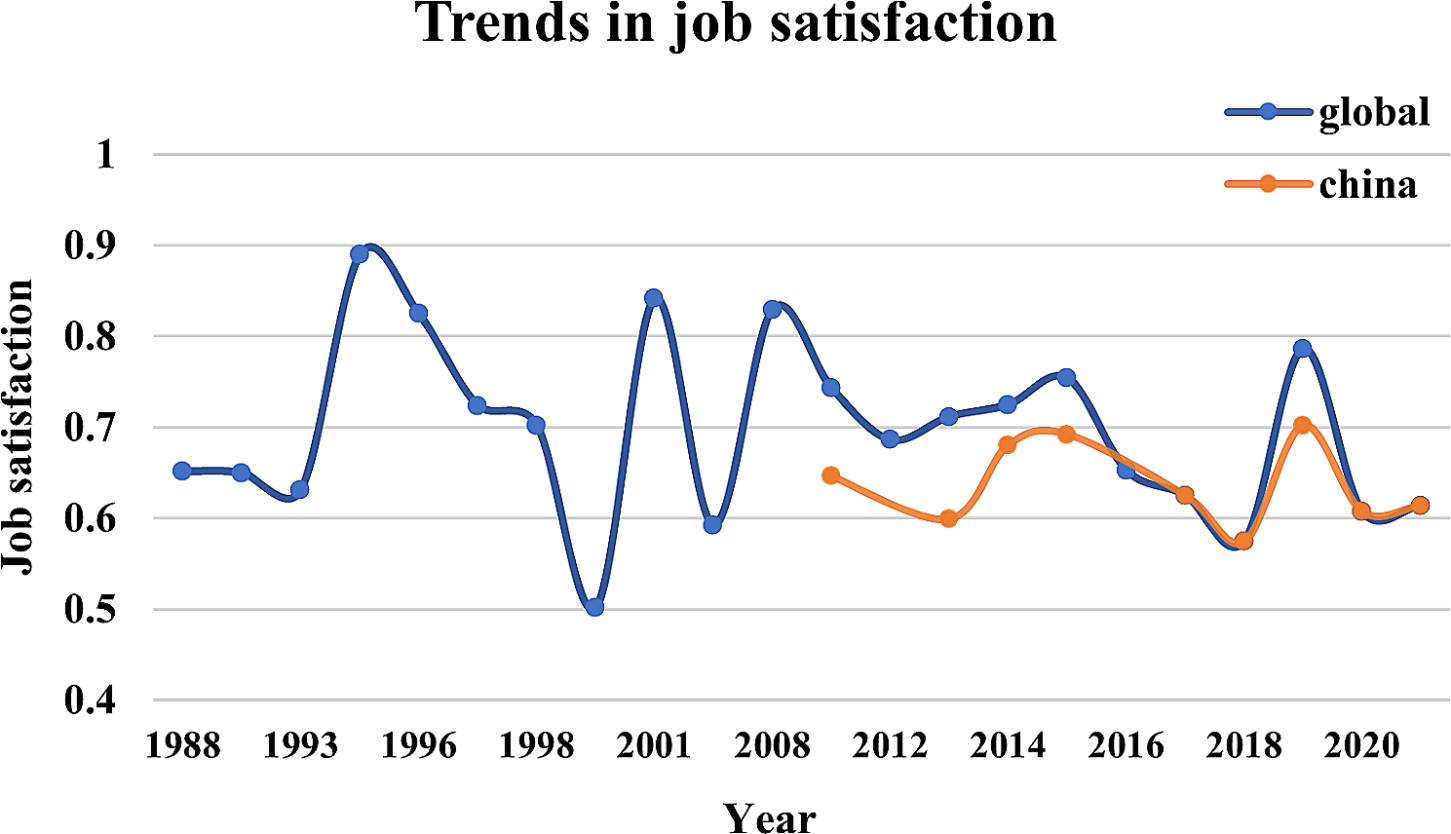
Trends in job satisfaction among CGPs

By further analyzing the studies in different years, we found that global CGPs’ satisfaction fluctuates up and down in the medium-upper stratum. In contrast, there has been a slight downward trend in recent years (Fig. 2). Besides, Chinese CGPs’ satisfaction has been lower than the global level in all years.

### Factors associated with job satisfaction

One hundred papers examined the factors influencing CGPs’ satisfaction. Based on previous studies [11, 12], we classified the influential factors into eight categories, namely personal traits, personal fulfilment, remuneration, job description, working conditions, governance, social recognition, and doctor-patient relationship. The breakdown of the factors is detailed in the Supplement 8.

Since the variety of reporting methods for measuring impact factors made it difficult to extract data, a comparative analysis of 100 papers was conducted (Table 3).

**Table 3:** Summary of research on factors influencing job satisfaction in CGPs

Region of study	Number of studies [n (%)]								
	Total	Factors							
		Personal traits	Personal fulfilment	Remuneration	Job description	working conditions	Governance	Social recognition	Doctor–patient relationship
China	48 (100.0)	34 (70.8)	30 (62.5)	40 (83.3)	29 (60.4)	22 (45.8)	28 (58.3)	11 (22.9)	6 (12.5)
Asia (Excluded China)	6 (100.0)	6 (100.0)	2 (33.3)	3 (50.0)	0 (0.0)	3 (50.0)	0 (0.0)	1 (16.7)	0 (0.0)
Europe	25 (100.0)	12 (48.0)	4 (16.0)	13 (52.0)	20 (80.0)	21 (84.0)	11 (44.0)	7 (28.0)	6 (24.0)
Middle East and Africa	5 (100.0)	5 (100.0)	4 (80.0)	4 (80.0)	2 (40.0)	3 (60.0)	2 (40.0)	1 (20.0)	1 (20.0)
North America and Oceania	16 (100.0)	10 (62.5)	3 (18.8)	9 (56.3)	12 (75.0)	15 (93.8)	4 (25.0)	2 (12.5)	3 (18.8)
Global	100 (100.0)	67 (67.0)	43 (43.0)	69 (69.0)	63 (63.0)	64 (64.0)	45 (45.0)	22 (22.0)	16 (16.0)

Note: CGPs, Community General Practitioners;

We found that remuneration was the most frequently meaningful factor amongst the 40 studies in China. This was followed by personal traits (34 studies, 70.8%) and personal fulfillment (30 studies, 62.5%). In the rest of Asia region, personal characteristics (6 studies, 100.0%) and remuneration (3 studies, 50.0%) were the most frequently cited factors contributing to CGPs’ satisfaction. In the Middle East and Africa region, every study noted the influence of personal traits (5 studies, 100.0%) on satisfaction. But working conditions were the most commonly stated factor in the Europe, North America and Oceania regions, with 21 (84.0%) and 15 (93.8%) reports.

In China, the factor that was least found to be statistically significant was doctor-patient relationship (6 studies, 12.5%). Concurrently, social recognition emerged as the least reported influential factor in regions such as the Middle East, Africa, North America, and Oceania.

Overall, remuneration (69 studies, 69.0%), personal traits (67 studies, 67.0%), and job description (63 studies, 63.0%) were noted the most frequently as statistically significant relationship with job satisfaction. Personal fulfillment (43 studies, 43.0%) and governance (45 studies, 45.0%) were reported in lower numbers, but still higher than social recognition (22 studies, 22.0%) and doctor-patient relationship (16 studies, 16.0%).

## Discussion

This article presents the first comprehensive summary and analysis of global literature pertaining to the job satisfaction of CGPs and its principal influencing factors, employing both meta-analysis and comparative analysis. The assessment results of job satisfaction and its influencing factors are vital references for current healthcare policies, which not only reflect the implementation effects of current CGPs policies, but also serve as one of the critical “wind vanes” for policy adjustment [13].

In China, Jing Feng et al. [14] showed that CGPs’ job satisfaction was an important factor influencing their turnover intention. Studies in other countries have reached the same conclusion. Ingris Gilled et al. [15] found that a decrease in overall job satisfaction reduces the willingness of healthcare workers to remain in their jobs. The results of a meta-analysis including 485 articles showed that job dissatisfaction was strongly associated with burnout, psychiatric problems, depression and anxiety [16]. Therefore, a comprehensive job satisfaction survey among CGPs is an essential factor in evaluating health policies and predicting turnover rates. However, the evidence for evaluating CGPs’ job satisfaction has been fragmented, either only within a particular country [17], only for rural doctors without considering CGPs in urban areas [11], or only a systematic review completed without meta-analysis [9]. This study not only collected the percentage of the number of satisfied CGPs globally, but also collected and integrated job satisfaction presented as continuous data using the Likert 5-level scale as a bridge and evaluated the overall job satisfaction after converting the formula. In addition, the results of studies from China and other countries on related influencing factors were compared and analyzed to provide basis for health care policymakers and community healthcare managers.

This study shows that the overall job satisfaction of CGPs is at a moderate level. As mentioned in the results, 3.52 (95% *CI*: 3.43–3.61) is the average satisfaction reported using the Likert 5-level scale. Based on the findings of the data presented in dichotomous form, it can be inferred that 70.82% (95% *CI*: 66.62–75.02%) of the CGPs who obtained the study were satisfied with their jobs. Nevertheless, we must carefully consider the credibility of the results due to the high degree of heterogeneity between studies. The publication year of the study and the income level of the country may be related to the observed heterogeneity.

The overall satisfaction level of CGPs in China is lower than the global average, but the gap with other regions is not obvious, which may be related to the income level of Chinese economy. According to the World Bank’s 2022 classification, China is in the upper-middle income level. In the subgroup analysis, 16 studies were conducted in the upper-middle-income region, and the percentage of CGPs who were satisfied with their jobs in this region (64.38%) was less than the other 28 CGPs who worked in the high-income region (74.49%). This is in line with the results reported in previous studies, where a cross-sectional study undertaken in 34 countries showed that the association between national economic level and satisfaction is considered universal, with the suggestion that national culture and institutions may contribute to this relationship [18].

Furthermore, this study found differences in satisfaction across time. The studies conducted during the coronavirus disease 2019 (COVID-19) pandemic (63.09%) were notably lower than those conducted during the period 2010–2019 (71.07%), and prior to 2009 (72.39%). These findings may indicate that CGPs have had to take on increasing responsibilities and multitasking during the prevention and control of COVID-19 pandemic, which placed a severe physical and psychological burden on primary care workers [19, 20]. Although some studies have shown that at the peak of the COVID-19 pandemic, 20-30% of healthcare workers reached critical levels of anxiety, depression and distress [21]. This study included only three articles conducted after the COVID-19 pandemic. Therefore, the interpretation of the results should be considered cautiously, and further extensive cohort studies may be needed to provide relevant evidence.

Notably, satisfaction among global CGPs has not been trending in a favorable direction over time and has even experienced a continued downward trend since the COVID-19 pandemic. As early as 2001, the BMJ [22] expressed concern about doctors’ job dissatisfaction, suggesting that they felt overworked and under-supported. CGPs feel intensely needed, while at the same time, financial overdrafts make it urgent to increase their income, which contributes to doctor burnout and a decline in the care quality [23–25]. Over the past two decades, several healthcare systems reform measures have been implemented in countries around the globe [26, 27]. Still, the results of the reforms are unsatisfactory considering the findings of this study. It is also worth reflecting on what will happen next if physician dissatisfaction continues to spread, and now may be an important point in time to consider the next strategies and changes that countries must implement.

Additionally, this study found a preference for reporting a significant impact from remuneration (79.6%) across Asia when exploring the factors influencing CGPs’ satisfaction. The Europe, North America, and Oceania regions were more concerned with the effects of job description and working conditions compared to the Asia region. We also found that overtime, work facilities, and work hours were more frequently identified as statistically significant factors than income (54.8%), accounting for more than 75% in these regions. This disparity is not only due to the variations in research perspectives across countries but also to the fact that CGPs have unique job demands and expectations depending on different national conditions, economic levels, and institutional attributes. It has been shown that wages are strongly correlated with job satisfaction in low-income countries, but the correlation does not exist in middle- and high-income countries [28]. Moreover, a large number of studies have shown that job autonomy has a significant effect on job satisfaction [29]. However, Chinese CGPs valued increased salary compensation more than hospital specialists. And their lower education level and lower demand for job autonomy may partially explain the differences in reporting of influencing factors from China and other countries [30, 31].

On the other hand, the workload problem of CGPs in China also deserves attention. In China, the shortage of CGPs has resulted in a heavier work burden and increased overtime work, which has triggered growth in dissatisfaction and higher turnover rates. Regarding work content, Chinese studies more often investigated the impact of management work on CGPs (29 studies, 60.4%). Some articles reported that CGPs need to invest considerable work time in tasks assigned by the public health sector since the National Health System in 2009. CGPs must spend much time and effort coping with excessively frequent performance appraisals [32]. Also, studies from other countries suggest that CGPs have more unnecessary non-health service tasks than doctors in other positions, which causes them to be more prone to burnout [33, 34].

Therefore, we suggest that the Government should prioritize the augmentation of funding community healthcare, with an initial focus on enhancing the compensation mechanisms for CGPs. Next, it is essential to carry out training work for the community health organizations’ personnel, conduct vocational training and quality education, improve the overall quality level, and create a favorable atmosphere of career development. Finally, work optimization based on the multi-professional teamwork model is conducive to higher efficiency and satisfaction levels.

There are some limitations of this study. First, the literature search lacked a global definition of the “community general practitioner” concept. Although the term’s meaning varies worldwide and healthcare systems are not entirely the same, there are sufficient similarities in working conditions to summarize and compare job satisfaction. Second, the diverse array of questionnaires and scales employed in the studies introduced a degree of variation that precluded the complete aggregation of results. However, this study included two types of data outcomes, both of which coherently reflected the general level of job satisfaction among CGPs. Third, in this study, the starting point of Chinese data is inconsistent with that of the global data, partly because China started to build the community health service system in 1997 before forming a genuinely community-based CGPs workforce. Another aspect is that early studies’ quality of CGPs’ job satisfaction needs to be improved based on literature review criteria. Furthermore, the reporting of influencing factors in the primary literature is reported through various methods and categories, making it difficult to extract data. As a result, it was difficult to combine the effects of the factors on job satisfaction. In the future, there is a need to use standardization to better identify the key factors that influence job satisfaction. Lastly, high heterogeneity collected among the studies may lead to a decrease in the credibility of the results.

## Conclusion

In this meta-analysis, the level of global job satisfaction among CGPs globally was moderate. Subgroup analyses found that global CGPs’ job satisfaction has not tended to increase over the years and may even decrease further over time. A comparison of changes in satisfaction over time reveals that Chinese CGPs’ satisfaction has been lower than the global level for many years. Additionally, the study highlighted regional variances in the factors influencing job satisfaction. In Asia, the primary focus of research is on salary and compensation, in contrast to the emphasis on working conditions predominant in studies from Europe and North America. Therefore, countries should pay more attention to the dissatisfaction of CGPs at work and take various measures to improve the career satisfaction of CGPs according to local conditions.

### Electronic supplementary material

Below is the link to the electronic supplementary material.

Supplementary Material 1

## Data Availability

The datasets supporting the conclusions of this article are included within the article and its supplement.

## Abbreviations

GP: General Practitioner
CGPs: General Practitioners
COVID-19: Coronavirus disease 2019
MSQ: Minnesota studies Questionnaire
WCW: Warr-Cook-Wall
CNKI: China National Knowledge Infrastructure
CASP: Critical Appraisal Skill Program
GNI: Gross National Income
