# Supplementary material for: Global job satisfaction and fluctuation among community general practitioners: a systematic review and meta-analysis
Source: BMC Health Serv Res. 2024 Mar 27;24:378. doi: 10.1186/s12913-024-10792-9 (PMC10967033; doi:10.1186/s12913-024-10792-9)
Supplement: Supplementary file 1 — Supplementary Material 1 [file 12913_2024_10792_MOESM1_ESM.docx]

**Supplementary Materials**

Supplementary 1: Search strategies 1

Supplementary 2: Screening process 2

Supplementary 3: Characteristics of included studies (n=129) 3

Supplementary 4: Quality evaluation of the studies 12

Supplementary 5: Forest plot 16

Supplementary 6: Funnel plot 17

Supplementary 7: Risk of bias plot 18

Supplementary 8: Classification of influencing factors 19

Supplementary 9: References for included studies 20

**Supplementary 1: Search strategies**

**PubMed: 14 June 2023**

("general practitioners"[MeSH Terms] OR general practitioners[Text Word]

"General Practitioners"[Mesh] OR "Physicians, Primary Care"[Mesh] OR "Physicians, Family"[Mesh]) AND "Job Satisfaction"[Mesh])

**Web of Science: 14 June 2023**

("general practitioners” OR "Physicians, Primary Care" OR "Physicians, Family") AND "Job Satisfaction"

Supplementary 2: Screening process

The study screening was performed through a three-step process. First, two authors (Q.D. and Y.L.) conducted a title-based screening, excluding studies that were explicitly unrelated to the study, such as intervention studies, studies with medical students or patient satisfaction as their subject matter. Studies excluded by all two authors were reviewed by the third author (J.L.) to resolve differences through discussion.

Second, after screening all abstract that were selected during step 1, the two authors followed the exclusion criteria to make a decision. All abstracts selected by each reviewer were discussed jointly to reach a consensus for inclusion or exclusion.

Third, Q.D. and Y.L. extracted data from each study included in the previous step.

Supplementary 3: Characteristics of included studies (n=129)

| Studies | Author's | Country | Region | Country income level | Publication years | Sampling method | Institutional Properties | Sample size | Tools used to measure satisfaction | Associated factors | Quality assessment score | |
| --- | --- | --- | --- | --- | --- | --- | --- | --- | --- | --- | --- | --- |
| 1 | Dowell, et al.(1) | Newzealand | NAAO | HIE | 2000 | RS | Non-public | 377 | WCW | 2、3、4、5、7 | 8 | |
| 2 | Allebdi, et al.(2) | Saudi Arabia | MEAA | HIE | 2020 | OS | Public | 119 | Paul et al. satisfaction scale | 1、2、3 | 5 | |
| 3 | Rihab, et al.(3) | Kuwait | MEAA | HIE | 2019 | OS | Public | 417 | WCW | 1、2、3 | 6 | |
| 4 | Cole, et al.(4) | America | NAAO | HIE | 2012 | NR | Public | 893 | Authors own questionnaire | 2、3、5、6 | 7 | |
| 5 | Scott, et al.(5) | England | Europe | HIE | 2006 | RS | NR | 2781 | WCW | 1、2、3、4、5、6、7 | 9 | |
| 6 | Sibbald, et al.(6) | England | Europe | HIE | 2000 | RS | Non-public | 4562 | WCW | NR | 9 | |
| 7 | Ulmer, et al.(7) | Australia | NAAO | HIE | 2002 | NR | NR | 406 | WCW | 1、5 | 6 | |
| 8 | Khalid, et al.(8) | Saudi Arabia | MEAA | HIE | 2018 | CS | Public | 237 | Authors own questionnaire | 1、2、5、6、7、8 | 7 | |
| 9 | Sibbald, et al.(9) | England | Europe | HIE | 2003 | RS | NR | 790 | WCW | 1、4、5 | 7 | |
| 10 | Sanchez-Piedra, et al.(10) | Europe | Europe | HIE | 2017 | SS | Non-public | 1331 | Authors own questionnaire | 1、3、4、5、6 | 7 | |
| 11 | Joyce, et al.(11) | Australia | NAAO | HIE | 2011 | NR | NR | 2223 | WCW | 1、3、4、5、8 | 7 | |
| 12 | Joyce, et al.(12) | Australia | NAAO | HIE | 2015 | OS | NR | 1078 | WCW | NR | 7 | |
| 13 | BH, et al.(13) | Malaysia | Asia | UMIE | 2013 | OS | Public | 149 | WCW | 1、5 | 7 | |
| 14 | Ifediora, et al.(14) | Australia | NAAO | HIE | 2016 | OS | NR | 142 | WCW | 1、3、4、5、6、7 | 7 | |
| 15 | Cohidon, et al.(15) | Europe | Europe | HIE | 2019 | RS | NR | 12049 | Authors own questionnaire | 1、4、5、6、8 | 8 | |
| 16 | Pathman, et al.(16) | America | NAAO | HIE | 1996 | NR | NR | 620 | Authors own questionnaire | 1、3、4、5 | 7 | |
| 17 | Wu, et al.(17) | China | Asia | UMIE | 2014 | MSPS | Public | 202 | THC satisfaction scale | 3、4、5、8 | 9 | |
| 18 | Whalley, et al.(18) | England | Europe | HIE | 2008 | RS | NR | 1349 | WCW | 3、4、6、8 | 7 | |
| 19 | French, et al.(19) | England | Europe | HIE | 2006 | RS | NR | 490 | WCW | NR | 6 | |
| 20 | Mazzaglia, et al.(20) | Italy | Europe | HIE | 2009 | NR | NR | 610 | WCW | 3 | 7 | |
| 21 | Alrawashdeh, et al.(21) | Jordan | Asia | UMIE | 2021 | NR | Public | 973 | SIJS | 1、3、5 | 6 | |
| 22 | Hayes, et al.(22) | England | Europe | HIE | 2020 | RS | NR | 2048 | WCW | NR | 8 | |
| 23 | Riisgaard, et al.(23) | [Denmark](link:Denmark) | Europe | HIE | 2017 | OS | Public | 1245 | COPSOQ | NR | 7 | |
| 24 | Ashraf, et al.(24) | Pakistan | Asia | LMIE | 2014 | OS | NR | 288 | Authors own questionnaire | 1、2、3、7 | 6 | |
| 25 | Jabbari, et al.(25) | [Iran](link:Iran) | Asia | LMIE | 2015 | OS | Public | 149 | WHO questionnaire | NR | 5 | |
| 26 | Jabbari, et al.(26) | [Iran](link:Iran) | Asia | LMIE | 2014 | OS | Public | 238 | WHO questionnaire | 1 | 7 | |
| 27 | Buciuniene, et al.(27) | [Lithuania](link:Lithuania) | Europe | HIE | 2005 | OS | Public | 243 | WCW | 3、4、5、6、7 | 6 | |
| 28 | Malhotra, et al.(28) | Canada | NAAO | HIE | 2018 | OS | NR | 32153 | Authors own questionnaire | 1、3、4、5 | 7 | |
| 29 | Soler, et al.(29) | Europe | Europe | HIE | 2008 | NR | NR | 1393 | Authors own questionnaire | NR | 9 | |
| 30 | Usta, et al.(30) | 49 countries | NAAO | HIE | 2022 | OS | NR | 205 | PWS | NR | 6 | |
| 31 | Gu, et al.(31) | China | Asia | UMIE | 2019 | SRS | Public | 495 | MSQ | 1、3、4、5、6 | 10 | |
| 32 | Chen, et al.(32) | China | Asia | UMIE | 2022 | RS | Public | 1105 | MSQ | 1、3、4、5 | 8 | |
| 33 | Szecsenyi, et al.(33) | Germany | Europe | HIE | 2011 | NR | Non-public | 676 | WCW | 4、5、8 | 7 | |
| 34 | Eaton-Hart, et al.(34) | England | Europe | HIE | 2022 | OS | NR | 2085 | WCW | NR | 7 | |
| 35 | Rosta, et al.(35) | Norway | Europe | HIE | 2019 | RS | Public | 219 | WCW | NR | 9 | |
| 36 | Charles, et al.(36) | [Denmark](link:Denmark) | Europe | HIE | 2018 | SRS | Public | 464 | Authors own questionnaire | 3、5、8 | 7 | |
| 37 | Nørøxe, et al.(37) | [Denmark](link:Denmark) | Europe | HIE | 2018 | OS | Public | 1697 | WCW | NR | 9 | |
| 38 | Goetz, et al.(38) | Germany | Europe | HIE | 2013 | RS | Non-public | 1027 | WCW | 1、4、5 | 8 | |
| 39 | Goetz, et al.(39) | Switzerland | Europe | HIE | 2016 | NR | Non-public | 176 | WCW | 1、4、5 | 6 | |
| 40 | Goetz, et al.(40) | Germany | Europe | HIE | 2018 | NR | Non-public | 470 | WCW | 1、3、4、5、7 | 6 | |
| 41 | Goetz, et al.(41) | Germany | Europe | HIE | 2011 | NR | Non-public | 643 | WCW | 3、4、5、7 | 6 | |
| 42 | Abdulrahman, et al.(42) | Saudi Arabia | MEAA | HIE | 2021 | NR | Public | 265 | Authors own questionnaire | NR | 7 | |
| 43 | Trevor, et al.(43) | England | Europe | HIE | 2013 | NR | NR | 2857 | Authors own questionnaire | NR | 8 | |
| 44 | Werdecker, et al.(44) | Germany | Europe | HIE | 2021 | SS | Non-public | 530 | WCW | 1、5 | 8 | |
| 45 | Li, et al.(45) | China | Asia | UMIE | 2014 | MRS | Public | 448 | MSQ | 1、3、4、5 | 8 | |
| 46 | Pedersen, et al.(46) | [Denmark](link:Denmark) | Europe | HIE | 2020 | OS | Public | 846 | COPSOQ | 5、7 | 7 | |
| 47 | Li, et al.(47) | China | Asia | UMIE | 2020 | MSRS | Public | 3236 | nurse professional identity scale | NR | 8 | |
| 48 | Degen, et al.(48) | Germany | Europe | HIE | 2021 | RS | Non-public | 366 | COPSOQ | NR | 6 | |
| 49 | Bass, et al.(49) | Canada | NAAO | HIE | 1998 | OS | NR | 190 | Authors own questionnaire | NR | 5 | |
| 50 | Daghio, et al.(50) | England | Europe | HIE | 2003 | NR | NR | 661 | Authors own questionnaire | NR | 7 | |
| 51 | Ramsbottom-Lucier, et al.(51) | America | NAAO | HIE | 1995 | OS | NR | 381 | Authors own questionnaire | 1、4、5 | 7 | |
| 52 | Nylenna, et al.(52) | Norway | Europe | HIE | 2005 | NR | Public | 295 | WCW | NR | 6 | |
| 53 | Lavanchy, et al.(53) | England | Europe | HIE | 2004 | RS | NR | 131 | WCW | 2、4、5、8 | 4 | |
| 54 | Behmann, et al.(54) | Germany | Europe | HIE | 2012 | RS | Non-public | 856 | Authors own questionnaire | 3、4、5、6、8 | 7 | |
| 55 | Harris, et al.(55) | Australia | NAAO | HIE | 2007 | NR | NR | 252 | WCW | 5 | 6 | |
| 56 | McGrail, et al.(56) | Australia | NAAO | HIE | 2012 | NR | NR | 3502 | WCW | 1、3、4、5 | 10 | |
| 57 | Pantell, et al.(57) | America | NAAO | HIE | 2019 | NR | NR | 890 | ISPCP | NR | 5 | |
| 58 | Andersen, et al.(58) | [Denmark](link:Denmark) | Europe | HIE | 2018 | OS | Public | 1906 | Authors own questionnaire | NR | 8 | |
| 59 | Zikusooka, et al.(59) | Turkey | Asia | UMIE | 2021 | OS | Public | 259 | MSQ | 1、3、5 | 7 | |
| 60 | Skolnik, et al.(60) | America | NAAO | HIE | 1993 | NR | NR | 1994 | Authors own questionnaire | NR | 6 | |
| 61 | Mrduljas-Dujic, et al.(61) | Croatia | Europe | HIE | 2010 | NR | Public | 131 | MOT-2004 | 2、3、4、5、6 | 8 | |
| 62 | Aasland, et al.(62) | Norway | Europe | HIE | 2010 | NR | Public | 1255 | WCW | NR | 8 | |
| 63 | Makin, et al.(63) | England | Europe | HIE | 1988 | RS | Non-public | 101 | WCW | 1、6 | 3 | |
| 64 | Chen, et al.(64) | America | NAAO | HIE | 2012 | SRS | Public | 1890 | Authors own questionnaire | 2、3、5、8 | 9 | |
| 65 | Chambers, et al.(65) | England | Europe | HIE | 1996 | NR | NR | 498 | WCW | NR | 4 | |
| 66 | Lepnurm, et al.(66) | Canada | NAAO | HIE | 1989 | RS | NR | 66 | Authors own questionnaire | 1、4、5 | 8 | |
| 67 | Leutgeb, et al.(67) | Germany | Europe | HIE | 2018 | OS | Non-public | 131 | WCW | 3、4、5、7 | 6 | |
| 68 | Christine, et al.(68) | Canada | NAAO | HIE | 2007 | OS | NR | 19726 | Authors own questionnaire | 1、4、5、6 | 8 | |
| 69 | Pillay, et al.(69) | South Africa | MEAA | UMIE | 2008 | RS | Non-public | 615 | PWLS | 1、3、4、5、6 | 7 | |
| 70 | Kalda, et al.(70) | Estonia | Europe | HIE | 2009 | NR | Non-public | 105 | WCW | 3、4、5、6 | 7 | |
| 71 | McGlone, et al.(71) | America | NAAO | HIE | 2001 | RS | NR | 353 | Authors own questionnaire | 1、4、6 | 6 | |
| 72 | Simoens, et al.(72) | England | Europe | HIE | 2002 | RS | Non-public | 1421 | WCW | 1、4、6 | 7 | |
| 73 | Deshpande, et al.(73) | America | NAAO | HIE | 2010 | SS | NR | 693 | Authors own questionnaire | 3、4、5、8 | 7 | |
| 74 | Bhattacherjee, et al.(74) | India | Asia | LMIE | 2016 | NR | Public | 255 | Kumar satisfaction scale | 1、2 | 4 | |
| 75 | Joos, et al.(75) | Germany | Europe | HIE | 2011 | RS | Non-public | 878 | WCW | NR | 8 | |
| 76 | Mercer, et al.(76) | England | Europe | HIE | 2023 | OS | NR | 479 | WCW | NR | 8 | |
| 77 | Kushnir, et al.(77) | [Israel](link:Israel) | MEAA | HIE | 1997 | OS | Non-public | 81 | Authors own questionnaire | 1、2、3、4、5 | 7 | |
| 78 | Gosden, et al.(78) | England | Europe | HIE | 2016 | RS | Non-public | 1828 | WCW | 1、4、5 | 5 | |
| 79 | Wen, et al.(79) | China | Asia | UMIE | 2018 | MSRS | Public | 440 | WCW | 2、3、4、5、6 | 8 | |
| 80 | Rout, et al.(80) | England | Europe | HIE | 1999 | RS | NR | 205 | WCW | NR | 7 | |
| 81 | Rout, et al.(81) | England | Europe | HIE | 1996 | RS | NR | 414 | WCW | 1、4、5、6 | 8 | |
| 82 | Rout, et al.(82) | England | Europe | HIE | 1994 | RS | Non-public | 2197 | WCW | 2、3、4、5、7 | 8 | |
| 83 | Cai, et al.(83) | China | Asia | UMIE | 2022 | MSRS | Public | 570 | MSQ | 1、3、4 | 9 | |
| 84 | Lin, et al.(84) | China | Asia | UMIE | 2020 | CS | Public | 1404 | Authors own questionnaire | NR | 8 | |
| 85 | Hueston, et al.(85) | America | NAAO | HIE | 1998 | RS | NR | 547 | Authors own questionnaire | 4、5 | 9 | |
| 86 | Yu, et al.(86) | China | Asia | UMIE | 2018 | SRS | Public | 850 | MSQ | 1 | 10 | |
| 87 | Zhang, et al.(87) | China | Asia | UMIE | 2016 | MSCS | Public | 935 | MSQ | 1、3、5、6、7、8 | 10 | |
| 88 | Jin, et al.(88) | China | Asia | UMIE | 2019 | CS | Public | 1146 | Authors own questionnaire | 3、4 | 7 | |
| 89 | Zhuo, et al.(89) | China | Asia | UMIE | 2020 | MSRS | Public | 2103 | MSQ | 1、2、3、4、7、8 | 10 | |
| 90 | Chang, et al.(90) | China | Asia | UMIE | 2016 | SRS | Public | 516 | JSS | 1、2、3、4 | 9 | |
| 91 | Chen, et al.(91) | China | Asia | UMIE | 2022 | MSCS | Public | 3301 | MSQ | 2、3、6 | 7 | |
| 92 | Chen, et al.(92) | China | Asia | UMIE | 2021 | SS | Public | 416 | MSQ | 1、2 | 9 | |
| 93 | Chen, et al.(93) | China | Asia | UMIE | 2022 | MSRS | Public | 101 | Song et al. satisfaction scale | 1、3 | 9 | |
| 94 | Chen, et al.(94) | China | Asia | UMIE | 2022 | RS | Public | 243 | Authors own questionnaire | 1、3、4 | 8 | |
| 95 | Feng, et al.(95) | China | Asia | UMIE | 2021 | MSRS | Public | 3236 | Shi et al. satisfaction scale | NR | 7 | |
| 96 | Han, et al.(96) | China | Asia | UMIE | 2023 | MSCRS | Public | 748 | Schreisheim satisfaction scale | 1、3、4、6、7 | 9 | |
| 97 | Hao, et al.(97) | China | Asia | UMIE | 2022 | MSCS | Public | 6006 | MSQ | 2、3 | 10 | |
| 98 | Huang, et al.(98) | China | Asia | UMIE | 2016 | SCS | Public | 79 | MSQ | 1、3、4、5、7 | 7 | |
| 99 | Huang, et al.(99) | China | Asia | UMIE | 2018 | MSRS | Public | 837 | Authors own questionnaire | 3、4、5、6、8 | 8 | |
| 100 | Jiao, et al.(100) | China | Asia | UMIE | 2020 | NR | Public | 235 | MSQ | 1、2 | 8 | |
| 101 | Jing, et al.(101) | China | Asia | UMIE | 2020 | MSRS | Public | 195 | Authors own questionnaire | 2、3、4、6 | 9 | |
| 102 | Kong, et al.(102) | China | Asia | UMIE | 2020 | RS | Public | 751 | MSQ | 1、2、3、4、5、6、7 | 8 | |
| 103 | Kong, et al.(103) | China | Asia | UMIE | 2021 | RS | Public | 219 | Authors own questionnaire | 2、3、4、5、6 | 7 | |
| 104 | Li, et al.(104) | China | Asia | UMIE | 2014 | CS | Public | 300 | MSQ | 1、2 | 9 | |
| 105 | Li, et al.(105) | China | Asia | UMIE | 2015 | CS | Public | 174 | MSQ | 2、3、5、6 | 8 | |
| 106 | Li, et al.(106) | China | Asia | UMIE | 2022 | SCS | Public | 159 | MSQ | 2、3、4、5、6、7 | 7 | |
| 107 | Liu, et al.(107) | China | Asia | UMIE | 2022 | MSRS | Public | 1727 | MSQ | 2、6 | 9 | |
| 108 | Luo, et al.(108) | China | Asia | UMIE | 2022 | NR | Public | 311 | MSQ | 1、2、3、4、5、6、7、8 | 9 | |
| 109 | Luo, et al.(109) | China | Asia | UMIE | 2015 | SRS | Public | 373 | Based on ACSI qustionnaire | 1、2、3、4、6 | 9 | |
| 110 | Ma, et al.(110) | China | Asia | UMIE | 2019 | NR | Public | 279 | MSQ | 2、3、4、5、6 | 9 | |
| 111 | Sun, et al.(111) | China | Asia | UMIE | 2017 | SS | Public | 215 | Greenhaus et al. scale | 3、5、6 | 8 | |
| 112 | Wang, et al.(112) | China | Asia | UMIE | 2023 | SRS | Public | 1229 | Song et al. satisfaction scale | 1、3、4、6 | 10 | |
| 113 | Wang, et al.(113) | China | Asia | UMIE | 2017 | NR | Public | 1159 | MSQ | 1 | 9 | |
| 114 | Wei, et al.(114) | China | Asia | UMIE | 2017 | SS | Public | 215 | Greenhaus et al. scale | NR | 9 | |
| 115 | Xing, et al.(115) | China | Asia | UMIE | 2010 | OS | Public | 2682 | MSQ | 1、2、3、4、6、7、8 | 8 | |
| 116 | Xu, et al.(116) | China | Asia | UMIE | 2017 | OS | Public | 983 | Authors own questionnaire | 1、2、3、5、6 | 8 | |
| 117 | Yang, et al.(117) | China | Asia | UMIE | 2017 | CS | Public | 152 | MSQ | 1、2、3、5、6、7 | 9 | |
| 118 | Yang, et al.(118) | China | Asia | UMIE | 2021 | CS | Public | 720 | MSQ | 1、2、3 | 9 | |
| 119 | Yang, et al.(119) | China | Asia | UMIE | 2016 | NR | Public | 206 | MSQ | 1、2 | 8 | |
| 120 | Yu, et al.(120) | China | Asia | UMIE | 2019 | SRS | Public | 3236 | Song et al. satisfaction scale | 1、2、3、5、6 | 9 | |
| 121 | Zhang, et al.(121) | China | Asia | UMIE | 2019 | SRS | Public | 382 | MSQ | 1、2、5 | 9 | |
| 122 | Zhang, et al.(122) | China | Asia | UMIE | 2020 | HS | Public | 1308 | MSQ | 1、2、3、4、5、6 | 9 | |
| 123 | Zhao, et al.(123) | China | Asia | UMIE | 2021 | SRS | Public | 388 | MSQ | 1、2、3、4、6 | 10 | |
| 124 | Zhao, et al.(124) | China | Asia | UMIE | 2022 | MSCRS | Public | 765 | Schreisheim satisfaction scale | 3、7 | 9 | |
| 125 | Zheng, et al.(125) | China | Asia | UMIE | 2020 | SS | Public | 251 | MSQ | 1、2、3、4、5、6、7 | 8 | |
| 126 | Zhou, et al.(126) | China | Asia | UMIE | 2020 | CS | Public | 6434 | Zhao et al. satisfaction scale | 1、2、3、4、6 | 9 | |
| 127 | Zhou, et al.(127) | China | Asia | UMIE | 2022 | SRS | Public | 229 | MSQ | 1、3、4、6 | 7 | |
| 128 | Zhou, et al.(128) | China | Asia | UMIE | 2019 | MSRS | Public | 1050 | Song et al. satisfaction scale | 1、2、3、4、6 | 9 | |
| 129 | Zhou, et al.(129) | China | Asia | UMIE | 2019 | SRS | Public | 338 | MSQ | 1、2、3、4、5、6 | 10 | |
| **Abbreviations:** NR, not reported; MEAA, Middle East And Africa; NAAO, North America And Oceania; LMIE, Lower-Middle-Income Economics; UMIE, Upper-Middle-Income Economics; HIE, High-Income Economics; | | | | | | | | | | | |  |
| **Sampling method:** MSRS, Multi-stage Stratified Random Sampling; MSPS, Multi-stage Stratified Purposive Sampling; MSCS, Multi-stage Stratified Cluster Sampling; MSCRS, Multi-stage Stratified Cluster Random Sampling; MRS, Multi-stage Random Sampling; CS, Cluster Sampling; SS, Stratified Sampling; SRS, Stratified Random Sampling; SCS, Stratified Cluster Sampling; OS, Overall Survey; RS, Random Sampling; PS, Purposive Sampling; HS, Handy Sampling； | | | | | | | | | | | |  |
| **Tools used to measure satisfaction**: WCW, Warr–Cook–Wall Job Satisfaction Scale; MSQ, Minnesota Job Satisfaction Questionnaire; JSS, Job Satisfaction Survey; WHO, World Health Organization (1993); SIJS, Short Index of Job Satisfaction; PWLS, Physician Work Life Survey (Williams et al., 1999; Konrad et al., 1999); THC, Township Health Center; PWS, Physician Worklife Survey; ISPSP, International Survey of Primary Care Physicians (2015); ACSI, American Customer Satisfaction Index; | | | | | | | | | | | |  |

Supplementary 4: Quality evaluation of the studies

| No. | Studies | D1 | D2 | D3 | D4 | D5 | D6 | D7 | D8 | D9 | D10 | D11 | Total | Assessment |
| --- | --- | --- | --- | --- | --- | --- | --- | --- | --- | --- | --- | --- | --- | --- |
| 1 | Dowell, et al. | 1 | 1 | 0 | 1 | 1 | 0 | 1 | 1 | 1 | 0 | 1 | 8 | High |
| 2 | Allebdi, et al. | 1 | 1 | 0 | 0 | 0 | 0 | 1 | Unclear | 1 | 0 | 1 | 5 | Medium |
| 3 | Rihab, et al. | 1 | 1 | 0 | 0 | 1 | 0 | 1 | Unclear | 1 | 0 | 1 | 6 | Medium |
| 4 | Cole, et al. | 1 | 1 | 1 | 0 | 0 | 0 | 1 | Unclear | 1 | 1 | 1 | 7 | Medium |
| 5 | Scott, et al. | 1 | 1 | 1 | 1 | 1 | 0 | 1 | 1 | 1 | 1 | 0 | 9 | High |
| 6 | Sibbald, et al. | 1 | 1 | 1 | 1 | 1 | 0 | 1 | 1 | 1 | 0 | 1 | 9 | High |
| 7 | Ulmer, et al. | 1 | 1 | 0 | 0 | 0 | 0 | 1 | 1 | 1 | 1 | 0 | 6 | Medium |
| 8 | Khalid, et al. | 1 | 1 | 1 | 0 | 0 | 0 | 1 | 1 | 1 | 1 | 0 | 7 | Medium |
| 9 | Sibbald, et al. | 1 | 1 | 1 | 1 | 0 | 0 | 1 | Unclear | 1 | 0 | 1 | 7 | Medium |
| 10 | Sanchez-Piedra, et al. | 1 | 1 | 1 | 1 | 0 | 0 | 0 | Unclear | 1 | 1 | 1 | 7 | Medium |
| 11 | Joyce, et al. | 1 | 1 | 1 | 1 | 0 | 0 | 1 | Unclear | 1 | 1 | 0 | 7 | Medium |
| 12 | Joyce, et al. | 1 | 1 | 1 | 1 | 0 | 0 | 1 | Unclear | 1 | 1 | 0 | 7 | Medium |
| 13 | BH, et al. | 1 | 1 | 0 | 1 | 0 | 0 | 1 | 1 | 1 | 1 | 0 | 7 | Medium |
| 14 | Ifediora, et al. | 1 | 1 | 0 | 1 | 0 | 0 | 1 | 1 | 1 | 1 | 0 | 7 | Medium |
| 15 | Cohidon, et al. | 1 | 1 | 1 | 1 | 0 | 0 | 1 | Unclear | 1 | 1 | 1 | 8 | High |
| 16 | Pathman, et al. | 1 | 1 | 1 | 0 | 0 | 0 | 1 | 1 | 1 | 1 | 0 | 7 | Medium |
| 17 | Wu, et al. | 1 | 1 | 1 | 1 | 1 | 0 | 1 | Unclear | 1 | 1 | 1 | 9 | High |
| 18 | Whalley, et al. | 1 | 1 | 1 | 0 | 0 | 0 | 1 | 1 | 1 | 1 | 0 | 7 | Medium |
| 19 | French, et al. | 1 | 1 | 0 | 0 | 0 | 0 | 1 | Unclear | 1 | 1 | 1 | 6 | Medium |
| 20 | Mazzaglia, et al. | 1 | 1 | 0 | 1 | 0 | 0 | 1 | 1 | 1 | 1 | 0 | 7 | Medium |
| 21 | Alrawashdeh, et al. | 1 | 1 | 0 | 0 | 0 | 1 | 0 | 1 | 1 | 1 | 0 | 6 | Medium |
| 22 | Hayes, et al. | 1 | 1 | 1 | 1 | 1 | 0 | 1 | Unclear | 1 | 1 | 0 | 8 | High |
| 23 | Riisgaard, et al. | 1 | 1 | 1 | 0 | 1 | 0 | 1 | Unclear | 1 | 1 | 0 | 7 | Medium |
| 24 | Ashraf, et al. | 1 | 1 | 0 | 0 | 0 | 0 | 1 | Unclear | 1 | 1 | 1 | 6 | Medium |
| 25 | Jabbari, et al. | 1 | 1 | 0 | 0 | 0 | 0 | 1 | 1 | 1 | 1 | 1 | 7 | Medium |
| 26 | Jabbari, et al. | 1 | 1 | 0 | 0 | 0 | 0 | 1 | Unclear | 1 | 0 | 1 | 5 | Medium |
| 27 | Buciuniene, et al. | 1 | 1 | 0 | 1 | 1 | 0 | 1 | Unclear | 1 | 0 | 0 | 6 | Medium |
| 28 | Malhotra, et al. | 1 | 1 | 0 | 1 | 0 | 0 | 1 | Unclear | 1 | 1 | 1 | 7 | Medium |
| 29 | Soler, et al. | 1 | 1 | 1 | 1 | 0 | 0 | 1 | 1 | 1 | 1 | 1 | 9 | High |
| 30 | Usta, et al. | 1 | 1 | 0 | 0 | 0 | 0 | 1 | 0 | 1 | 1 | 1 | 6 | Medium |
| 31 | Gu, et al. | 1 | 1 | 1 | 1 | 1 | 0 | 1 | 1 | 1 | 1 | 1 | 10 | High |
| 32 | Chen, et al. | 1 | 1 | 1 | 1 | 1 | 0 | 1 | Unclear | 1 | 0 | 1 | 8 | High |
| 33 | Szecsenyi, et al. | 1 | 1 | 1 | 0 | 1 | 0 | 1 | Unclear | 1 | 1 | 0 | 7 | Medium |
| 34 | Eaton-Hart, et al. | 1 | 1 | 1 | 1 | 1 | 0 | 1 | Unclear | 1 | 0 | 0 | 7 | Medium |
| 35 | Rosta, et al. | 1 | 1 | 1 | 1 | 1 | 0 | 1 | 1 | 1 | 1 | 0 | 9 | High |
| 36 | Charles, et al. | 1 | 1 | 1 | 0 | 0 | 0 | 1 | Unclear | 1 | 1 | 1 | 7 | Medium |
| 37 | Nørøxe, et al. | 1 | 1 | 1 | 1 | 1 | 0 | 1 | 1 | 1 | 1 | 0 | 9 | High |
| 38 | Goetz, et al. | 1 | 1 | 1 | 0 | 0 | 0 | 1 | Unclear | 1 | 0 | 1 | 6 | Medium |
| 39 | Goetz, et al. | 1 | 1 | 1 | 1 | 0 | 0 | 1 | 1 | 1 | 1 | 0 | 8 | High |
| 40 | Goetz, et al. | 1 | 1 | 0 | 0 | 0 | 0 | 1 | Unclear | 1 | 1 | 1 | 6 | Medium |
| 41 | Goetz, et al. | 1 | 1 | 1 | 0 | 0 | 0 | 1 | 0 | 1 | 1 | 0 | 6 | Medium |
| 42 | Abdulrahman, et al. | 1 | 1 | 0 | 0 | 1 | 1 | 1 | Unclear | 1 | 0 | 1 | 7 | Medium |
| 43 | Trevor, et al. | 1 | 1 | 1 | 1 | 1 | 0 | 1 | Unclear | 1 | 0 | 1 | 8 | High |
| 44 | Werdecker, et al. | 1 | 1 | 1 | 0 | 0 | 1 | 1 | 1 | 1 | 1 | 0 | 8 | High |
| 45 | Li, et al. | 1 | 1 | 1 | 1 | 0 | 0 | 1 | 1 | 1 | 1 | 0 | 8 | High |
| 46 | Pedersen, et al. | 1 | 1 | 1 | 1 | 0 | 0 | 1 | Unclear | 1 | 1 | 0 | 7 | Medium |
| 47 | Li, et al. | 1 | 1 | 1 | 1 | 1 | 0 | 1 | Unclear | 1 | 1 | 0 | 8 | High |
| 48 | Degen, et al. | 1 | 0 | 1 | 0 | 0 | 1 | 1 | Unclear | 1 | 0 | 1 | 6 | Medium |
| 49 | Bass, et al. | 1 | 1 | 1 | 0 | 0 | 0 | 1 | Unclear | 1 | 0 | 0 | 5 | Medium |
| 50 | Daghio, et al. | 1 | 1 | 1 | 0 | 1 | 1 | 1 | Unclear | 1 | 0 | 0 | 7 | Medium |
| 51 | Ramsbottom-Lucier, et al. | 1 | 1 | 0 | 1 | 0 | 0 | 1 | 1 | 1 | 1 | 0 | 7 | Medium |
| 52 | Nylenna, et al. | 1 | 1 | 1 | 0 | 1 | 0 | 1 | Unclear | 1 | 0 | 0 | 6 | Medium |
| 53 | Lavanchy, et al. | 1 | 1 | 0 | 0 | 0 | 0 | 1 | 1 | 0 | 0 | 0 | 4 | Medium |
| 54 | Behmann , et al. | 1 | 1 | 1 | 1 | 0 | 0 | 1 | Unclear | 1 | 0 | 1 | 7 | Medium |
| 55 | Harris, et al. | 1 | 1 | 0 | 0 | 0 | 0 | 1 | 1 | 1 | 1 | 0 | 6 | Medium |
| 56 | McGrail, et al. | 1 | 1 | 1 | 1 | 1 | 0 | 1 | 1 | 1 | 1 | 1 | 10 | High |
| 57 | Pantell, et al. | 1 | 1 | 0 | 0 | 0 | 0 | 1 | Unclear | 1 | 1 | 0 | 5 | Medium |
| 58 | Andersen, et al. | 1 | 1 | 1 | 1 | 1 | 0 | 1 | Unclear | 1 | 1 | 0 | 8 | High |
| 59 | Zikusooka, et al. | 1 | 1 | 1 | 1 | 0 | 0 | 1 | Unclear | 1 | 1 | 0 | 7 | Medium |
| 60 | Skolnik, et al. | 1 | 1 | 1 | 0 | 0 | 0 | 1 | Unclear | 1 | 0 | 1 | 6 | Medium |
| 61 | Mrduljas-Dujic, et al. | 1 | 1 | 0 | 0 | 1 | 0 | 1 | 1 | 1 | 1 | 1 | 8 | High |
| 62 | Aasland, et al. | 1 | 1 | 1 | 1 | 1 | 0 | 1 | 1 | 0 | 1 | 0 | 8 | High |
| 63 | Makin, et al. | 1 | 1 | 1 | 0 | 0 | 0 | 1 | Unclear | 0 | 0 | 0 | 4 | Medium |
| 64 | Chen, et al. | 1 | 1 | 1 | 1 | 1 | 0 | 1 | Unclear | 1 | 1 | 1 | 9 | High |
| 65 | Chambers, et al. | 1 | 1 | 0 | 0 | 0 | 0 | 1 | Unclear | 1 | 0 | 0 | 4 | Medium |
| 66 | Lepnurm, et al. | 1 | 1 | 1 | 1 | 1 | 0 | 1 | 1 | 1 | 0 | 0 | 8 | High |
| 67 | Leutgeb, et al. | 1 | 1 | 0 | 0 | 0 | 0 | 1 | 1 | 1 | 1 | 0 | 6 | Medium |
| 68 | Christine, et al. | 1 | 1 | 1 | 0 | 1 | 0 | 1 | Unclear | 1 | 1 | 1 | 8 | High |
| 69 | Pillay, et al. | 1 | 1 | 1 | 1 | 0 | 0 | 1 | Unclear | 1 | 1 | 0 | 7 | Medium |
| 70 | Kalda, et al. | 1 | 1 | 0 | 1 | 0 | 0 | 1 | 1 | 1 | 1 | 0 | 7 | Medium |
| 71 | McGlone, et al. | 1 | 1 | 0 | 0 | 0 | 0 | 1 | 1 | 1 | 1 | 0 | 6 | Medium |
| 72 | Simoens, et al. | 1 | 1 | 1 | 0 | 0 | 0 | 1 | 1 | 1 | 0 | 1 | 7 | Medium |
| 73 | Deshpande, et al. | 1 | 1 | 1 | 1 | 0 | 0 | 0 | 1 | 1 | 0 | 1 | 7 | Medium |
| 74 | Bhattacherjee, et al. | 1 | 1 | 0 | 0 | 0 | 0 | 0 | Unclear | 0 | 1 | 1 | 4 | Medium |
| 75 | Joos, et al. | 1 | 1 | 1 | 1 | 0 | 0 | 1 | 1 | 1 | 1 | 0 | 8 | High |
| 76 | Mercer, et al. | 1 | 1 | 1 | 1 | 1 | 0 | 1 | Unclear | 1 | 0 | 1 | 8 | High |
| 77 | Kushnir, et al. | 1 | 1 | 0 | 1 | 1 | 0 | 1 | 1 | 1 | 0 | 0 | 7 | Medium |
| 78 | Gosden, et al. | 1 | 1 | 1 | 1 | 0 | 0 | 0 | Unclear | 1 | 0 | 0 | 5 | Medium |
| 79 | Wen, et al. | 1 | 1 | 1 | 1 | 0 | 0 | 1 | 1 | 1 | 0 | 1 | 8 | High |
| 80 | Rout, et al. | 1 | 1 | 1 | 1 | 0 | 0 | 1 | 1 | 1 | 0 | 1 | 8 | High |
| 81 | Rout, et al. | 1 | 1 | 1 | 0 | 0 | 0 | 1 | 1 | 1 | 1 | 1 | 8 | High |
| 82 | Rout, et al. | 1 | 1 | 1 | 0 | 0 | 0 | 1 | 1 | 1 | 1 | 0 | 7 | Medium |
| 83 | Cai, et al. | 1 | 1 | 1 | 1 | 0 | 0 | 1 | 1 | 1 | 1 | 1 | 9 | High |
| 84 | Lin, et al. | 1 | 1 | 1 | 0 | 0 | 0 | 1 | 1 | 1 | 1 | 1 | 8 | High |
| 85 | Hueston, et al. | 1 | 1 | 1 | 1 | 1 | 0 | 1 | Unclear | 1 | 1 | 1 | 9 | High |
| 86 | Yu, et al. | 1 | 1 | 1 | 1 | 0 | 1 | 1 | 1 | 1 | 1 | 1 | 10 | High |
| 87 | Zhang, et al. | 1 | 1 | 1 | 1 | 1 | 0 | 1 | 1 | 1 | 1 | 1 | 10 | High |
| 88 | Jin, et al. | 1 | 1 | 1 | 1 | 0 | 0 | 0 | Unclear | 1 | 1 | 1 | 7 | Medium |
| 89 | Zhuo, et al. | 1 | 1 | 1 | 1 | 1 | 0 | 1 | 1 | 1 | 1 | 1 | 10 | High |
| 90 | Chang, et al. | 1 | 1 | 1 | 1 | 1 | 0 | 1 | 1 | 1 | 0 | 1 | 9 | High |
| 91 | Chen, et al. | 1 | 1 | 1 | 1 | 0 | 0 | 0 | 1 | 1 | 0 | 1 | 7 | Medium |
| 92 | Chen, et al. | 1 | 1 | 1 | 1 | 1 | 0 | 1 | 1 | 1 | 0 | 1 | 9 | High |
| 93 | Chen, et al. | 1 | 1 | 1 | 1 | 1 | 0 | 1 | 1 | 1 | 0 | 1 | 9 | High |
| 94 | Chen, et al. | 1 | 1 | 1 | 1 | 1 | 0 | 1 | 1 | 1 | 0 | 0 | 8 | High |
| 95 | Feng, et al. | 1 | 1 | 1 | 1 | 1 | 0 | 1 | Unclear | 1 | 0 | 0 | 7 | Medium |
| 96 | Han, et al. | 1 | 1 | 1 | 1 | 1 | 0 | 1 | 1 | 1 | 0 | 1 | 9 | High |
| 97 | Hao, et al. | 1 | 1 | 1 | 1 | 1 | 0 | 1 | 1 | 1 | 1 | 1 | 10 | High |
| 98 | Huang, et al. | 1 | 1 | 1 | 0 | 1 | 0 | 1 | Unclear | 1 | 0 | 1 | 7 | Medium |
| 99 | Huang, et al. | 1 | 1 | 1 | 1 | 1 | 0 | 1 | 1 | 1 | 0 | 0 | 8 | High |
| 100 | Jiao, et al. | 1 | 1 | 1 | 0 | 1 | 0 | 1 | 1 | 1 | 0 | 1 | 8 | High |
| 101 | Jing, et al. | 1 | 1 | 1 | 1 | 1 | 0 | 1 | Unclear | 1 | 1 | 1 | 9 | High |
| 102 | Kong, et al. | 1 | 1 | 1 | 1 | 1 | 0 | 1 | Unclear | 1 | 0 | 1 | 8 | High |
| 103 | Kong, et al. | 1 | 1 | 1 | 0 | 1 | 0 | 1 | Unclear | 1 | 1 | 0 | 7 | Medium |
| 104 | Li, et al. | 1 | 1 | 1 | 1 | 1 | 0 | 1 | 1 | 1 | 0 | 1 | 9 | High |
| 105 | Li, et al. | 1 | 1 | 1 | 1 | 0 | 0 | 1 | 1 | 1 | 0 | 1 | 8 | High |
| 106 | Li, et al. | 1 | 1 | 1 | 1 | 1 | 0 | 1 | Unclear | 1 | 0 | 0 | 7 | Medium |
| 107 | Liu, et al. | 1 | 1 | 1 | 1 | 1 | 0 | 1 | 1 | 1 | 0 | 1 | 9 | High |
| 108 | Luo, et al. | 1 | 1 | 1 | 0 | 1 | 0 | 1 | 1 | 1 | 1 | 1 | 9 | High |
| 109 | Luo, et al. | 1 | 1 | 1 | 1 | 1 | 0 | 1 | 1 | 1 | 1 | 0 | 9 | High |
| 110 | Ma, et al. | 1 | 1 | 1 | 1 | 1 | 0 | 1 | 1 | 1 | 0 | 1 | 9 | High |
| 111 | Sun, et al. | 1 | 1 | 1 | 1 | 1 | 0 | 1 | 1 | 1 | 0 | 0 | 8 | High |
| 112 | Wang, et al. | 1 | 1 | 1 | 1 | 1 | 0 | 1 | 1 | 1 | 1 | 1 | 10 | High |
| 113 | Wang, et al. | 1 | 1 | 1 | 1 | 1 | 0 | 1 | 1 | 1 | 1 | 0 | 9 | High |
| 114 | Wei, et al. | 1 | 1 | 1 | 1 | 1 | 0 | 1 | 1 | 1 | 0 | 1 | 9 | High |
| 115 | Xing, et al. | 1 | 1 | 1 | 0 | 1 | 0 | 1 | Unclear | 1 | 1 | 1 | 8 | High |
| 116 | Xu, et al. | 1 | 1 | 1 | 0 | 1 | 0 | 1 | 1 | 1 | 0 | 1 | 8 | High |
| 117 | Yang, et al. | 1 | 1 | 1 | 1 | 1 | 0 | 1 | 1 | 1 | 0 | 1 | 9 | High |
| 118 | Yang, et al. | 1 | 1 | 1 | 1 | 1 | 0 | 1 | 1 | 1 | 0 | 1 | 9 | High |
| 119 | Yang, et al. | 1 | 1 | 1 | 0 | 1 | 0 | 1 | 1 | 1 | 0 | 1 | 8 | High |
| 120 | Yu, et al. | 1 | 1 | 1 | 1 | 1 | 0 | 1 | 1 | 1 | 0 | 1 | 9 | High |
| 121 | Zhang, et al. | 1 | 1 | 1 | 1 | 1 | 0 | 1 | 1 | 1 | 0 | 1 | 9 | High |
| 122 | Zhang, et al. | 1 | 1 | 1 | 0 | 1 | 0 | 1 | 1 | 1 | 1 | 1 | 9 | High |
| 123 | Zhao, et al. | 1 | 1 | 1 | 1 | 1 | 0 | 1 | 1 | 1 | 1 | 1 | 10 | High |
| 124 | Zhao, et al. | 1 | 1 | 1 | 1 | 1 | 0 | 1 | 1 | 1 | 0 | 1 | 9 | High |
| 125 | Zheng, et al. | 1 | 1 | 1 | 0 | 1 | 0 | 1 | Unclear | 1 | 1 | 1 | 8 | High |
| 126 | Zhou, et al. | 1 | 1 | 1 | 1 | 1 | 0 | 1 | 1 | 1 | 0 | 1 | 9 | High |
| 127 | Zhou, et al. | 1 | 1 | 1 | 1 | 1 | 0 | 1 | Unclear | 1 | 0 | 0 | 7 | Medium |
| 128 | Zhou, et al. | 1 | 1 | 1 | 1 | 1 | 0 | 1 | Unclear | 1 | 1 | 1 | 9 | High |
| 129 | Zhou, et al. | 1 | 1 | 1 | 1 | 1 | 0 | 1 | 1 | 1 | 1 | 1 | 10 | High |
| D1, Did the study address a clearly focused question/ issue? | | | | | | | | | | | | | | |
| D2, Is the research method (study design) appropriate for answering the research question? | | | | | | | | | | | | | | |
| D3, Is the method of selection of the subjects (employees, teams, divisions, organizations) clearly described? | | | | | | | | | | | | | | |
| D4, Could the way the sample was obtained introduce (selection) bias? | | | | | | | | | | | | | | |
| D5, Was the sample of subjects representative with regard to the population to which the findings will be referred? | | | | | | | | | | | | | | |
| D6, Was the sample size based on pre-study considerations of statistical power? | | | | | | | | | | | | | | |
| D7, Was a satisfactory response rate achieved? | | | | | | | | | | | | | | |
| D8, Are the measurements (questionnaires) likely to be valid and reliable? | | | | | | | | | | | | | | |
| D9, Was the statistical significance assessed? | | | | | | | | | | | | | | |
| D10, Are confidence intervals given for the main results? | | | | | | | | | | | | | | |
| D11, Could there be confounding factors that haven’t been accounted for? | | | | | | | | | | | | | | |


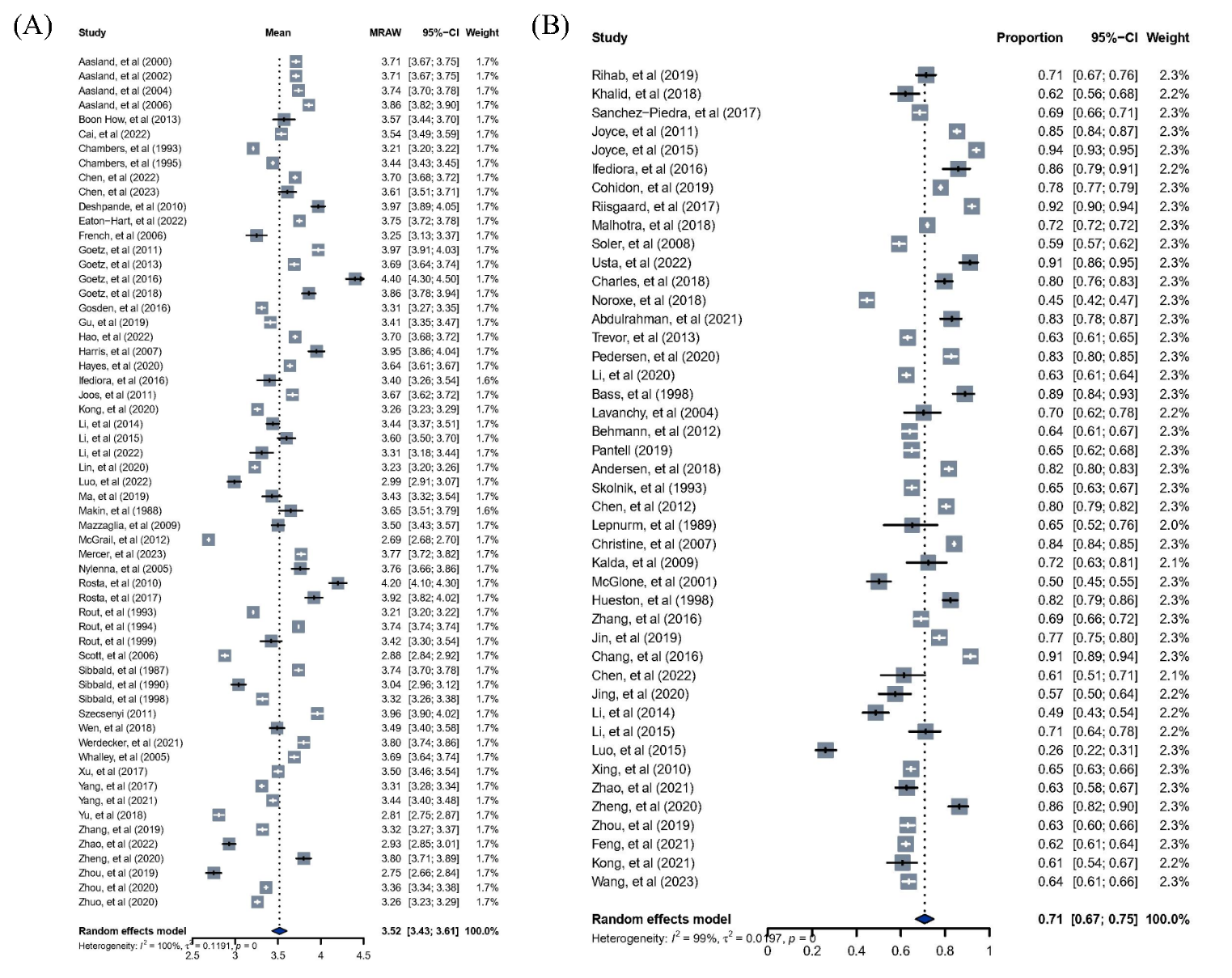
Supplementary 5: Forest plot

Supplementary 6: Funnel plot


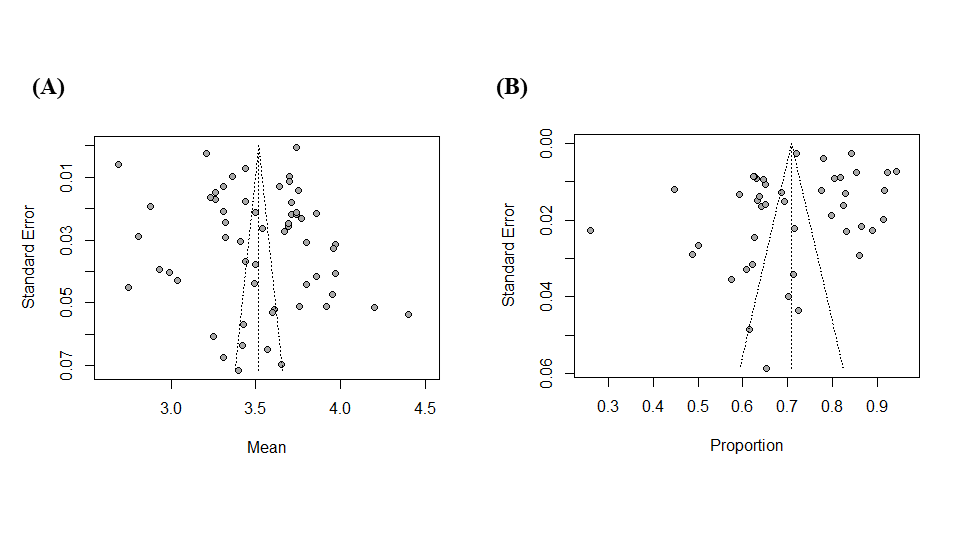


Supplementary 7: Risk of bias plot


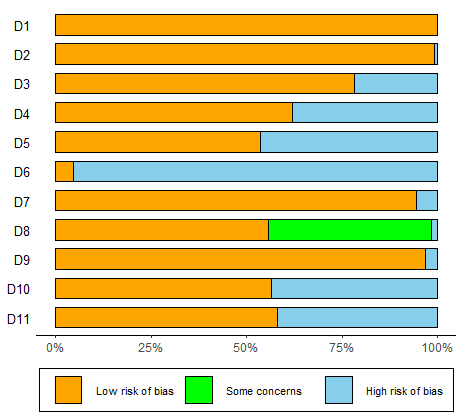


Supplementary 8: Classification of influencing factors

|  | Theme | Factors |
| --- | --- | --- |
| 1 | Personal traits | Gender, age, marriage status, race, education level, working years, work-life balance, mental health, raise child under the age of 18, live with family; |
| 2 | Personal fulfilment | Career development opportunities, job title, achievement, advancement, opportunity to utilize capabilities; |
| 3 | Remuneration | Salary、bonus, benefits; |
| 4 | Job description | Work stress, work intensity, workload, job autonomy (Clinical Decision-making Freedom), work variety, average weekly working hours, weekly overtime hours, job security; |
| 5 | working conditions | Colleague support, size of the institution, work location (urban/rural), working environment, resource allocation; |
| 6 | Governance | Supervisor support, administrative tasks, training opportunities, institutional management system; |
| 7 | Social recognition | Social status, social respect； |
| 8 | Doctor–patient relationship | Issues in doctor-patient communication; Having sufficient time to care for patients, doctor-patient relationship |

Supplementary 9: References for included studies

1. A. C. Dowell, S. Hamilton, D. K. McLeod. Job satisfaction, psychological morbidity and job stress among New Zealand general practitioners. N Z Med J. 2000 Jul 14;113(1113):269–72.

2. AbdulLateef A. Allebdi, Hanan M. Ibrahim. Level and determinants of job satisfaction among Saudi physicians working in primary health-care facilities in Western Region, KSA. J Fam Med Prim CARE. 2020 Sep;9(9):4656–61.

3. Al Wotayan Rihab, Annaka Mahmoud, Nazar Maqsood. Job Satisfaction and Mental Health among Physicians in Primary Health Care Centers in Kuwait. Health (N Y). 2019;(06 vo 11).

4. Allison M. Cole, Mark Doescher, William R. Phillips, Paul Ford, Nancy G. Stevens. Satisfaction of family physicians working in community health centers. J Am Board Fam Med JABFM. 2012 Aug;25(4):470–6.

5. Anthony Scott, Hugh Gravelle, Steven Simoens, Chris Bojke, Bonnie Sibbald. Job satisfaction and quitting intentions: A structural model of British general practitioners. Br J Ind Relat. 2006 Sep;44(3):519–40.

6. B. Sibbald, I. Enzer, C. Cooper, U. Rout, V. Sutherland. GP job satisfaction in 1987, 1990 and 1998: lessons for the future? Fam Pract. 2000 Oct;17(5):364–71.

7. Barbara Ulmer, Mark Harris. Australian GPs are satisfied with their job: even more so in rural areas. Fam Pract. 2002 Jun;19(3):300–3.

8. Bawakid Khalid, Rashid Ola Abdul, Mandoura Najlaa, Shah Hassan Bin Usman, Mugharbel Kholood. Professional satisfaction of family physicians working in primary healthcare centers: A comparison of two Saudi regions. J Fam Med Prim Care. 2018;(5 vo 7).

9. Bonnie Sibbald, Chris Bojke, Hugh Gravelle. National survey of job satisfaction and retirement intentions among general practitioners in England. BMJ. 2003 Jan 4;326(7379):22–4.

10. Carlos Alberto Sanchez-Piedra, Lina Jaruseviciene, Francisco Javier Prado-Galbarro, Ida Liseckiene, Fernando Sánchez-Alonso, Sonia García-Pérez, et al. Factors associated with professional satisfaction in primary care: Results from EUprimecare project. Eur J Gen Pract. 2017 Dec;23(1):114–20.

11. Catherine M. Joyce, Stefanie Schurer, Anthony Scott, John Humphreys, Guyonne Kalb. Australian doctors’ satisfaction with their work: results from the MABEL longitudinal survey of doctors. Med J Aust. 2011 Jan 3;194(1):30–3.

12. Catherine M. Joyce, Wei C. Wang, Hayley M. McDonald. Retirement patterns of Australian doctors aged 65 years and older. Aust Health Rev. 2015;39(5):582–7.

13. Chew BH, Ramli AS, Omar M, Ismail IZ. A preliminary study of job satisfaction and motivation among the Malaysian primary healthcare professionals. Malays Fam Physician. 2013;(2 vo 8).

14. Chris O. Ifediora. Assessing the satisfaction levels among doctors who embark on after-hours home visits in Australia. Fam Pract. 2016 Feb;33(1):82–8.

15. Christine Cohidon, Pascal Wild, Nicolas Senn. Practice Organization Characteristics Related to Job Satisfaction Among General Practitioners in 11 Countries. Ann Fam Med. 2019 Dec;17(6):510–7.

16. D. E. Pathman, E. S. Williams, T. R. Konrad. Rural physician satisfaction: its sources and relationship to retention. J Rural Health Off J Am Rural Health Assoc Natl Rural Health Care Assoc. 1996 Fall;12(5):366–77.

17. Dan Wu, Yun Wang, Kwok Fai Lam, Therese Hesketh. Health system reforms, violence against doctors and job satisfaction in the medical profession: a cross-sectional survey in Zhejiang Province, Eastern China. BMJ OPEN. 2014;4(12).

18. Diane Whalley, Hugh Gravelle, Bonnie Sibbald. Effect of the new contract on GPs’ working lives and perceptions of quality of care: a longitudinal study. Br J Gen Pract. 2008 Jan;58(546):8–14.

19. Fiona French, Jane Andrew, Morag Awramenko, Helen Coutts, Linda Leighton-Beck, Jill Mollison, et al. Why do work patterns differ between men and women GPs? J Health Organ Manag. 2006;20(2–3):163–72.

20. Giampiero Mazzaglia, Francesco Lapi, Caterina Silvestri, Lorenzo Roti, Saffi Ettore Giustini, Eva Buiatti. Association between satisfaction and stress with aspects of job and practice management among primary care physicians. Qual Prim Care. 2009;17(3):215–23.

21. Hamzeh Mohammad Alrawashdeh, Ala’a B. Al-Tammemi, Mohammad Kh Alzawahreh, Ashraf Al-Tamimi, Mohamed Elkholy, Fawaz Al Sarireh, et al. Occupational burnout and job satisfaction among physicians in times of COVID-19 crisis: a convergent parallel mixed-method study. BMC PUBLIC Health. 2021 Apr 28;21(1).

22. Helen Hayes, Jonathan Gibson, Bridie Fitzpatrick, Kath Checkland, Bruce Guthrie, Matt Sutton, et al. Working lives of GPs in Scotland and England: cross-sectional analysis of national surveys. BMJ Open. 2020 Oct 30;10(10):e042236.

23. Helle Riisgaard, Jens Søndergaard, Maria Munch, Jette V. Le, Loni Ledderer, Line B. Pedersen, et al. Associations between degrees of task delegation and job satisfaction of general practitioners and their staff: a cross-sectional study. BMC Health Serv Res. 2017 Jan 17;17(1):44.

24. Hiba Ashraf, Nasir Shah, Fahad Anwer, Hina Akhtar, Mairaj Anwar Abro, Asma Khan. Professional satisfaction of family physicians in Pakistan--results of a cross-sectional postal survey. JPMA J Pak Med Assoc. 2014 Apr;64(4):442–6.

25. Hossein Jabbari, Mohammad Zakarria Pezeshki, Mohammad Naghavi Behzad, Mohammad Asghari, Reza Piri, Fariba Bakhshian. What is the relationship between family physicians’ job satisfaction associated and their educational performance? J Anal Res Clin Med. 2015;(1 vo 3).

26. Hossein Jabbari, Mohamad Zakarria Pezeshki, Mohammad Naghavi-Behzad, Mohammad Asghari, Fariba Bakhshian. Relationship between job satisfaction and performance of primary care physicians after the family physician reform of east Azerbaijan province in Northwest Iran. Indian J Public Health. 2014 Dec;58(4):256–60.

27. Ilona Buciuniene, Aurelija Blazeviciene, Egle Bliudziute. Health care reform and job satisfaction of primary health care physicians in Lithuania. BMC Fam Pract. 2005 Mar 7;6(1):10.

28. Jana Malhotra, Eric Wong, Amardeep Thind. Canadian family physician job satisfaction - is it changing in an evolving practice environment? An analysis of the 2013 National Physician Survey database. BMC Fam Pract. 2018 Jun 23;19(1):100.

29. Jean Karl Soler, Hakan Yaman, Magdalena Esteva, Frank Dobbs, Radost Spiridonova Asenova, Milica Katic, et al. Burnout in European family doctors: the EGPRN study. Fam Pract. 2008 Aug;25(4):245–65.

30. Jinan Usta, Lucy M. Candib, Sonia Oyola, Farah Abdul Razzak, Jumana Antoun. Women family physicians’ working conditions and career satisfaction: a multinational study. Fam Pract. 2022 May 28;39(3):323–31.

31. Jingliang Gu, Tianmin Zhen, Yan Song, Lingzhong Xu. Job satisfaction of certified primary care physicians in rural Shandong Province, China: a cross-sectional study. BMC Health Serv Res. 2019 Jan 28;19(1):75.

32. Jinhua Chen, Yijun Wang, Wen Du, Shuyi Liu, Zhu Xiao, Yuelei Wu. Analysis on the relationship between effort-reward imbalance and job satisfaction among family doctors in China: a cross-sectional study. BMC Health Serv Res. 2022 Aug 4;22(1):992.

33. Joachim Szecsenyi, Katja Goetz, Stephen Campbell, Bjoern Broge, Bernd Reuschenbach, Michel Wensing. Is the job satisfaction of primary care team members associated with patient satisfaction? BMJ Qual Saf. 2011 Jun;20(6):508–14.

34. Jonathan H. Eaton-Hart, John Cm Gillies, Stewart W. Mercer. How do the working lives of general practitioners in rural areas compare with elsewhere in Scotland? Cross-sectional analysis of the Scottish School of Primary Care National GP Survey. Rural Remote Health. 2022 Jul;22(3):7270.

35. Judith Rosta, Olaf G. Aasland, Magne Nylenna. Changes in job satisfaction among doctors in Norway from 2010 to 2017: a study based on repeated surveys. BMJ OPEN. 2019 Sep;9(9).

36. Justin A. Charles, Peder Ahnfeldt-Mollerup, Jens Søndergaard, Troels Kristensen. Empathy Variation in General Practice: A Survey among General Practitioners in Denmark. Int J Environ Res Public Health. 2018 Mar 2;15(3).

37. Karen Busk Nørøxe, Anette Fischer Pedersen, Flemming Bro, Peter Vedsted. Mental well-being and job satisfaction among general practitioners: a nationwide cross-sectional survey in Denmark. BMC Fam Pract. 2018 Jul 28;19(1):130.

38. Katja Goetz, Berthold Musselmann, Joachim Szecsenyi, Stefanie Joos. The influence of workload and health behavior on job satisfaction of general practitioners. Fam Med. 2013 Feb;45(2):95–101.

39. Katja Goetz, Marianne Jossen, Joachim Szecsenyi, Thomas Rosemann, Karolin Hahn, Sigrid Hess. Job satisfaction of primary care physicians in Switzerland: an observational study. Fam Pract. 2016 Oct;33(5):498–503.

40. Katja Goetz, Janis Mahnkopf, Anna Kornitzky, Jost Steinhäuser. Difficult medical encounters and job satisfaction - results of a cross sectional study with general practitioners in Germany. BMC Fam Pract. 2018 May 9;19(1):57.

41. Katja Goetz, Stephen M. Campbell, Jost Steinhaeuser, Bjoern Broge, Sara Willms, Joachim Szecsenyi. Evaluation of job satisfaction of practice staff and general practitioners: an exploratory study. BMC Fam Pract. 2011 Dec 12;12:137–137.

42. Khalid Bin Abdulrahman, Moath Yosef Alnosian, Abdulwahab Ali Alshamrani, Hatim Ibrahim ALassaf, Abdulrahman Saleh Aldayel, Yazeed Ahmed Alaskar, et al. Job satisfaction among family medicine physicians in Saudi Arabia. J Fam Med Prim CARE. 2021 Aug 27;10(8):2952–7.

43. Lambert Trevor, Smith Fay, Goldacre Michael. GPs’ job satisfaction: doctors who chose general practice early or late. Br J Gen Pract J R Coll Gen Pract. 2013;(616 vo 63).

44. Lena Werdecker, Tobias Esch. Burnout, satisfaction and happiness among German general practitioners (GPs): A cross-sectional survey on health resources and stressors. PloS One. 2021 Jun 18;16(6):e0253447.

45. Li Li, Zhong Zhang, Zhinan Sun, Hao Zhou, Xinyan Liu, Heng Li, et al. Relationships between actual and desired workplace characteristics and job satisfaction for community health workers in China: a cross-sectional study. BMC Fam Pract. 2014 Nov 18;15:180.

46. Line Bjørnskov Pedersen, Thomas Allen, Frans Boch Waldorff, Merethe Kirstine Kousgaard Andersen. Does accreditation affect the job satisfaction of general practitioners? A combined panel data survey and cluster randomised field experiment. Health Policy Amst Neth. 2020 Aug;124(8):849–55.

47. Liqing Li, Yong Gan, Yudi Yang, Heng Jiang, Kai Lu, Xiaogang Zhou, et al. Analysis on professional identity and related factors among Chinese general practitioners: a National Cross-sectional Study. BMC Fam Pract. 2020 May 6;21(1):80.

48. Lukas Degen, Karen Linden, Tanja Seifried-Dübon, Brigitte Werners, Matthias Grot, Esther Rind, et al. Job Satisfaction and Chronic Stress of General Practitioners and Their Teams: Baseline Data of a Cluster-Randomised Trial (IMPROVEjob). Int J Environ Res Public Health. 2021 Sep 8;18(18):9458.

49. M. J. Bass, I. R. McWhinney, M. Stewart, A. Grindrod. Changing face of family practice. Can Fam Physician Med Fam Can. 1998 Oct;44:2143–9.

50. M. M. Daghio, A. V. Ciardullo, T. Cadioli, C. Delvecchio, A. Menna, C. Voci, et al. GPs’ satisfaction with the doctor-patient encounter: findings from a community-based survey. Fam Pract. 2003 Jun;20(3):283–8.

51. M. T. Ramsbottom-Lucier, T. S. Caudill, M. M. Johnson, E. C. Rich. Interactions with colleagues and their effects on the satisfaction of rural primary care physicians. J Rural Health Off J Am Rural Health Assoc Natl Rural Health Care Assoc. 1995 Summer;11(3):185–91.

52. Magne Nylenna, Pål Gulbrandsen, Reidun Førde, Olaf G. Aasland. Job satisfaction among Norwegian general practitioners. Scand J Prim Health Care. 2005 Dec;23(4):198–202.

53. Marcel Lavanchy, Ian Connelly, Stefan Grzybowski, Alex C. Michalos, Jonathan Berkowitz, Harvey V. Thommasen. Determinants of Rural Physicians’ Life and Job Satisfaction. Soc Indic Res. 2004;(1 vo 69).

54. Mareike Behmann, Guido Schmiemann, Heidrun Lingner, Franziska Kühne, Eva Hummers-Pradier, Nils Schneider. Job satisfaction among primary care physicians: results of a survey. Dtsch Arzteblatt Int. 2012 Mar 16;109(11):193–200.

55. Mark F. Harris, Judy G. Proudfoot, Upali W. Jayasinghe, Christine H. Holton, Gawaine P. Powell Davies, Cheryl L. Amoroso, et al. Job satisfaction of staff and the team environment in Australian general practice. Med J Aust. 2007 Jun 4;186(11):570–3.

56. Matthew R. McGrail, John S. Humphreys, Catherine M. Joyce, Anthony Scott. International medical graduates mandated to practise in rural Australia are highly unsatisfied: Results from a national survey of doctors. Health Policy. 2012;(2–3 vo 108).

57. Matthew S. Pantell, Emilia De Marchis, Angeli Bueno, Laura M. Gottlieb. Practice Capacity to Address Patients’ Social Needs and Physician Satisfaction and. Perceived. Quality of Care. Ann Fam Med. 2019 Feb;17(1):42–5.

58. Merethe Kirstine Andersen, Line Bjørnskov Pedersen, Frans Boch Waldorff. Retirement, job satisfaction and attitudes towards mandatory accreditation: a Danish survey study in general practice. BMJ Open. 2018 Aug 13;8(8):e020419.

59. Monica Zikusooka, Omur Cinar Elci, Habibe Özdemir. Job satisfaction among Syrian healthcare workers in refugee health centres. Hum Resour Health. 2021 Nov 14;19(1):140.

60. N. S. Skolnik, D. R. Smith, J. Diamond. Professional satisfaction and dissatisfaction of family physicians. J Fam Pract. 1993 Sep;37(3):257–63.

61. Natasa Mrduljas-Dujic, Marion Kuzmanic, Goran Kardum, Mirjana Rumboldt. Job Satisfaction among Medical Doctors in One of the Countries in Transition: Experience from Croatia. Coll Antropol. 2010 Sep;34(3):813–8.

62. Olaf G. Aasland, Judith Rosta, Magne Nylenna. Healthcare reforms and job satisfaction among doctors in Norway. Scand J Public Health. 2010 May;38(3):253–8.

63. P. J. Makin, U. Rout, C. L. Cooper. Job satisfaction and occupational stress among general practitioners--a pilot study. J R Coll Gen Pract. 1988 Jul;38(312):303–6.

64. Peggy G. Chen, Leslie A. Curry, Marcella Nunez-Smith, Elizabeth H. Bradley, Mayur M. Desai. Career satisfaction in primary care: a comparison of international and US medical graduates. J Gen Intern Med. 2012 Feb;27(2):147–52.

65. R. Chambers, D. Wall, I. Campbell. Stresses, coping mechanisms and job satisfaction in general practitioner registrars. Br J Gen Pract J R Coll Gen Pract. 1996 Jun;46(407):343–8.

66. R. Lepnurm, M. Trowell. Satisfaction of Country Doctors. Healthc Manage Forum. 1989 Fall;2(3):14–20.

67. R. Leutgeb, J. Frankenhauser-Mannuß, M. Scheuer, J. Szecsenyi, Katja Goetz. Job satisfaction and stressors for working in out-of-hours care – a pilot study with general practitioners in a rural area of Germany. BMC Fam Pract. 2018;(1 vo 19).

68. Rivet Christine, Ryan Bridget, Stewart Moira. Hands on: is there an association between doing procedures and job satisfaction? Can Fam Physician Med Fam Can. 2007;(1 vo 53).

69. Rubin Pillay. Work satisfaction of medical doctors in the South African private health sector. J Health Organ Manag. 2008;22(3):254–68.

70. Ruth Kalda, Maaroos, Lember. Motivation and satisfaction among Estonian family doctors working in different settings. Eur J Gen Pract. 2000;(1 vo 6).

71. S J McGlone, LG Chenoweth. Job demands and control as predictors of occupational satisfaction in general practice. Med J Aust. 2001 Jul 16;175(2):88-+.

72. S. Simoens, A. Scott, B. Sibbald. Job satisfaction, work-related stress and intentions to quit of Scottish GPS. Scott Med J. 2002 Aug;47(4):80–6.

73. Satish P. Deshpande, Jim Demello. An empirical investigation of factors influencing career satisfaction of primary care physicians. J Am Board Fam Med JABFM. 2010 Dec;23(6):762–9.

74. Sharmistha Bhattacherjee, Kuntala Ray, Jayanta Kumar Roy, Abhijit Mukherjee, Hironmoy Roy, Saikat Datta. Job Satisfaction among Doctors of a Government Medical College and Hospital of Eastern India. Nepal J Epidemiol. 2016 Oct;6(3):596–602.

75. Stefanie Joos, Berthold Musselmann, Joachim Szecsenyi, Katja Goetz. Characteristics and job satisfaction of general practitioners using complementary and alternative medicine in Germany - is there a pattern? BMC Complement Altern Med. 2011 Dec 19;11:131.

76. Stewart W. Mercer, Carey J. Lunan, Clare MacRae, David Ag Henderson, Bridie Fitzpatrick, John Gillies, et al. Half a century of the inverse care law: A comparison of general practitioner job satisfaction and patient satisfaction in deprived and affluent areas of Scotland. Scott Med J. 2023 Feb;68(1):14–20.

77. T Kushnir, S Melamed, J Ribak. Occupational physicians in Israel: Work structure, job and personal characteristics, and job satisfaction. J Occup Environ Med. 1997 Sep;39(9):874–81.

78. Toby Gosden, Jacky Williams, Roland Petchey, Brenda Leese, Bonnie Sibbald. Salaried contracts in UK general practice: a study of job satisfaction and stress. J Health Serv Res Policy. 2002 Jan;7(1):26–33.

79. Tong Wen, Yan Zhang, Xue Wang, Guo Tang. Factors influencing turnover intention among primary care doctors: a cross-sectional study in Chongqing, China. Hum Resour Health. 2018 Feb 13;16(1):10.

80. U. Rout. Job stress among general practitioners and nurses in primary care in England. Psychol Rep. 1999 Dec;85(3 Pt 1):981–6.

81. U. Rout, Cary L. Cooper, Jaya K. Rout. Job stress among British general practitioners: Predictors of job dissatisfaction and mental ill-health. Stress Med. 1996;12(3):155–66.

82. U. Rout, J K Rout. Job satisfaction, mental health and job stress among general practitioners before and after the new contract--a comparative study. Fam Pract. 1994 Sep;11(3):300–6.

83. Weiqin Cai, Yuanze Du, Qianqian Gao, Runguo Gao, Hongqing An, Wenwen Liu, et al. Satisfaction of family physician team members in the context of contract system: A cross-sectional survey from Shandong Province, China. Front Public Health. 2022 Nov 25;10:1000299.

84. Wei-Quan Lin, Le-Xin Yuan, Shi-Yuan Kuang, Xiao-Xia Zhang, Can-Jie Lu, Ting-Ting Lin, et al. Work engagement as a mediator between organizational commitment and job satisfaction among community health-care workers in China: a cross-sectional study. Psychol Health Med. 2020 Jul 2;25(6):666–74.

85. William J. Hueston. Family physicians’ satisfaction with practice. Arch Fam Med. 1998 Jun;7(3):242–7.

86. Xiaoge Yu, Mengyun Zheng, Xiaoqing Cheng, Bing Xu, Zhao Tao, Jiehui Ding, et al. Job Satisfaction Among Doctors from Jiangsu Province in China. Med Sci Monit Int Med J Exp Clin Res. 2018 Oct 8;24:7162–9.

87. Xiaoyan Zhang, Pengqian Fang. Job satisfaction of village doctors during the new healthcare reforms in China. Aust Health Rev Publ Aust Hosp Assoc. 2016 Apr;40(2):225–33.

88. Yinzi Jin, Haipeng Wang, Dan Wang, Beibei Yuan. Job satisfaction of the primary healthcare providers with expanded roles in the context of health service integration in rural China: a cross-sectional mixed methods study. Hum Resour Health. 2019 Sep 2;17(1):70.

89. Zhang Zhuo, Shi Guoshuai, Li Lingui, Bian Ying. Job satisfaction among primary care physicians in western China. BMC Fam Pract. 2020;(1 vo 21).

90. Xuan Chang, Tianai Yin. Study on the Relationship among Job Satisfaction,Career Burnout and Intent to Stay in General Practitioners [Internet] [master]. Shandong University; 2015. Available from: https://d.wanfangdata.com.cn/thesis/ChJUaGVzaXNOZXdTMjAyMzAxMTISCFkyNzkwNzg0GghjY3N2cDRlOA%3D%3D

91. Chutian Chen, Donghua Wan, Aihua Hao. Study on job satisfaction of general practitioners in primary medical and health institutions in Pearl River Delta. Chin J Public Health Manag. 2022;(05 vo 38):621-624+632.

92. Dongming Chen, Weicun Ren, Yanjie Wang, Jia Li, Qingfeng Tian. Surveying the Satisfaction Degree of Grassroots General Practitioners in Henan Province. Chin Health Serv Manag. 2021;38(03 vo 38):212–5.

93. Dongran Chen, Peilan Xu, Lei Ding, Liuming Ding, Yuhua Li. Analysis of the status quoand influencing factors of job satisfaction of grassroots general practitioners in Urumqi. China Mod Dr. 2022;60(26 vo 60):92–6.

94. Jin Chen, Hongshu Hao, Hui Tao, Zhenfeng Gao, Caiyong Li, Jiawen Tan, et al. Job Satisfaction and Influencing Factors of Family Doctors in Chongqing City. Vol. 29, Chinese Health Quality Management. 2022. p. 66–71.

95. Jing Feng, Xin Shen, Jianxiong Wu, Yilin Lou, Yong Gan. Status Quo and Influencing Factors of Professional Identity Among General Practitioners in China. Vol. 34, Medicine and Society. 2021. p. 13–6.

96. Wantong Han, Yurong Jing, Wenzhe Qin, Fangfang Hu, Jiao Zhang, Zhaorong Gao, et al. Study on Influencing Factors of Family Doctors’ Job Satisfaction in Tai’an Based on Motivation-hygiene Theory. Med Soc. 2023;(01 vo 36):29-33+44.

97. Aihua Hao, Chutian Chen, Donghua Wan, Qun He. Level and Associated Factors of Job Satisfaction in General Practitioners in Guangdong’s Primary Care Settings. Vol. 25, Chinese General Practice. 2022. p. 1629–35.

98. Jiaoling Huang, Hong Liang, Yimin Zhang, Xinguo Wang, Weisheng Zhang, Jianmin Zhang, et al. Analysis of influencing factors of family doctors’ satisfaction. Vol. 19, Chinese Health Resources. 2016. p. 507–11.

99. Shusheng Huang, Liqing Zhang, Qianqian Liu, TIecheng Liu, Xiaoyuan Qu, Aitian Yin. Analysis of job satisfaction and its related factors of general practitioners in Shandong Province. Vol. 21, Chinese Health Resources. 2018. p. 257–61.

100. Jie Jiao, Hongbao Wang. Investigation and Association Analysis of Job Satisfaction,Psychological Capital and Work Engagement of General Practitioners in Jiading District of Shanghai. Vol. 23, Chinese General Practice. 2020. p. 1144–50.

101. Zhengyue Jing, Yuejing Feng, Lulu Ding, Xue Tang, Chengchao Zhou. Job satisfaction of family doctors in rural Shandong, China and influencing factors: A study from the perspective of health providers. Vol. 40, Chinese Rural Health Service Administration. 2020. p. 230–4.

102. Guoshu Kong, Minghang Zhong, Fukang Zhang. An empirical research on the influence of social demographic characteristics on family doctors’ job satisfaction in primary healthcare institutions——Evidence based on survey data from three provinces located in Central and Western regions of China. Vol. 13, Chinese Journal of Health Policy. 2020. p. 49–56.

103. Hong Kong, Yixi Zhang, Xiujuan Zhang. Career satisfaction survey of community general practitioners in Ningbo City. Vol. 41, Chinese Rural Health Service Administration. 2021. p. 793–7.

104. Junrong Li, Lei Li, Yang Sun. Research on Job Satisfaction and related Influencing Factors among Staff in Community Health Service Center. Mod Prev Med. 2014;41(14):2557–9.

105. Shujie Li, Hairui Zhang, Lina Zhu, Yu Qin, Wenyuan Ma, Li Ma. Status Quo of Occupational Pressure and Job Satisfaction of General Practitioners and Its Influencing Factor. Vol. 18, Chinese General Practice. 2015. p. 387–90.

106. Xinwei Li, Lianyu Han, Huayi Zhang, Hua Zhang. Survey on practice status and job satisfaction of general practitioners in primary medical institutions, Jinan city. Prev Med Trib. 2022;28(04 vo 28):264–8.

107. Junlin Liu, Gong Feng, Zhiqian Du, Na He. Mediating Role of Job Satisfaction in the Relationship between Job Satisfaction and Career Development Satisfaction in Healthcare Workers of Township Hospitals. Vol. 25, Chinese General Practice. 2022. p. 3537–43.

108. Xiao Luo, Xi He, Haibing Li, Li Tu, Hailing Zhang, Lin Yao, et al. Study on career satisfaction and related factors of “5+3” directional general practitioner in Guizhou Province. Vol. 36, Soft Science of Health. 2022. p. 92–6.

109. Da Luo, Wenxiu Yang, Chao Han. Satisfaction Degree of General Practitioners in Tianjin With Their Practice and Its Influencing Factors. Chinese General Practice. 2015. p. 2701–4.

110. Tianjiao Ma, Jinghua Li, Li Zhang, Ruijie Zhang, Qian Zhang. Job Satisfaction and Influencing Factors of Medical Staff in a Community in Changchun City Under Family Doctor Contract Service Mode. Vol. 32, Medicine and Society. 2019. p. 30-33+51.

111. Tao Sun, Qiuru Cao, Shue Zhang, Yan Zhao, Fan Chao, Li Li, et al. The influence factors of career satisfaction of general practitioner in city ’s community health service institution. Vol. 15, Chinese Journal of General Practice. 2017. p. 183–5.

112. Hongwei Wang, Ziqin Deng, Wenjun Ping, Hao Dong, Xinyu Zhang, Wu He, et al. Analysis of Job Satisfaction and Its Influencing Factors of General Practitioners in Eastern China. Chin J Soc Med. 2023;40(1):76–80.

113. Jian Wang, Qi Zhao, Li Yuan, Zhigang Pan. Survey on work satisfaction among general practitioners in Shanghai. Vol. 16, Chinese Journal of General Practitioners. 2017. p. 921–5.

114. Hongjuan Wei, Qingdong Luo, Shue Zhang, Yu Shi, Fengzhe Xie, Shuo Wang, et al. Correlation of Organizational Ownership Behavior with Job Satisfaction and the Mediating Effect of Job Embeddedness between Them in General Practitioners. Vol. 20, Chinese General Practice. 2017. p. 1539-1542+1548.

115. Liying Xing, Xueliu Lu, Xiaofeng Tian, He Qiu, Wei Guo, Lixia He, et al. A Survey of Influencing Factors to Rural Doctors’ Satisfaction in Liaoning Province. Chin Gen Pract. 2010;13(4):397–8.

116. Bin Xu, Xiaping Huang, Biling Jiang. Job Satisfaction of Primary Medical Workers in Nanning City. J Prev Med Inf. 2017;33(9):859–64.

117. Mingrong Yang. Survey of Job Satisfaction of General Practitioner in Jilin Area. China Health Ind. 2017;(31 vo 14):5–7.

118. Shiyu Yang. Study on Job Satisfaction of Family Doctors from the Perspective of Psychological Contract——Take Hubei Province as an Example. Chin Masters Theses Full-Text Database. 2021;

119. Wei Yang, Sunfang Jiang. Job Satisfaction Survey of Young and Middle-aged General Practitioner in Community Healthcare Center and Comparison with Specialist Doctors’ [Internet] [Master’s Theses]. FuDan University; 2014. Available from: https://d.wanfangdata.com.cn/thesis/ChJUaGVzaXNOZXdTMjAyMzAxMTISCFkyODY3NTg5GghjY3N2cDRlOA%3D%3D

120. Fang Yu, Yanling Zheng, Tingting Yang, Yudi Yang, Jianxin Liu, Chao Wang, et al. Job satisfaction and its influencing factors among general practitioners in the eastern, central, and western China. Mod Prev Med. 2019;46(16 vo 46):2986–90.

121. Tingjian Zhang. Investigation on Job Satisfaction of General Practitioners——Taking Anhui Province as an Example. Chin Health Serv Manag. 2019;(06 vo 36):422-424+442.

122. Xi Zhang, Weinan Liu, Binhai Zhu. A Study about job satisfaction and its influencing factors of general practitioners in Jiangsu province. Vol. 31, Jiangsu Healthcare Administration. 2020. p. 1493–8.

123. Jing Zhao, Shengran Fan, Xiaofeng Shi, Xinghua Huo, Changhong Yan. Analysis of Job Satisfaction and Related Factors about Family Doctors Based on Supplier Perspective. Vol. 35, Chinese Primary Health Care. 2021. p. 14–6.

124. Yan Zhao, Lingzhong Xu, Wenzhe Qin, Fangfang Hu, Wantong Han, Yurong Jing, et al. Current Situation and Correlation Analysis Between Perceived Social Support and Job Satisfaction of Family Doctors in Tai’an. Vol. 35, Medicine and Society. 2022. p. 75–9.

125. Chanjiao Zheng, Baoxin Chen, Zhijie Huang, Huanyu Shen, Weilin Ou, Dongying Zhang, et al. Job Satisfaction and Influencing Factors in the First Batch of Community Health Workers Delivered Pilot Family Doctor Services in Guangdong Province. Vol. 23, Chinese General Practice. 2020. p. 964–8.

126. Dishu Zhou, Yongzhu Xu, Lin Wei, Renping Tian, Lihua Yi, JIngfu Qiu. Study on work stress, job satisfaction and turnover intention of primary medical staffs in Chongqing. Mod Prev Med. 2020;47(14):2545-2549,2574.

127. Tongru Zhou, Qiaoli Wang, Ping Liu. Job Satisfaction of General Practitioners and Its Multifactor Analysis in Yinchuan. Vol. 17, China Journal of Pharmaceutical Economics. 2022. p. 21-23+28.

128. Xiaogang Zhou, Shuilin Chen, Liqing Li, Yong Gan. Job Satisfaction and Influencing Factors among General Practitioners in Western China. Vol. 22, Chinese General Practice. 2019. p. 4266–71.

129. Wanjun Zou, Shaoqun Zhang, Fang Gong. Job Satisfaction and Associated Factors among General Practitioners from Primary Healthcare Institutions in Chongqing. Vol. 22, Chinese General Practice. 2019. p. 3819–24.
